# Transmission of Nonconjugative Virulence or Resistance Plasmids Mediated by a Self-Transferable IncN3 Plasmid from Carbapenem-Resistant Klebsiella pneumoniae

**DOI:** 10.1128/spectrum.01364-22

**Published:** 2022-07-14

**Authors:** Xiaoli Wang, Bin Tang, Guitian Liu, Meng Wang, Jingyong Sun, Ruoming Tan, Tingting Pan, Jieming Qu, Jialin Liu, Hong-Yu Ou, Hongping Qu

**Affiliations:** a Department of Critical Care Medicine, Ruijin Hospital, Shanghai Jiao Tong University School of Medicine, Shanghai, China; b State Key Laboratory of Microbial Metabolism, Shanghai-Islamabad-Belgrade Joint Innovation Center on Antibacterial Resistances, Joint International Laboratory on Metabolic & Developmental Sciences, School of Life Sciences & Biotechnology, Shanghai Jiao Tong University, Shanghai, China; c Department of Clinical Microbiology, Ruijin Hospital, Shanghai Jiao Tong University School of Medicine, Shanghai, China; d Department of Pulmonary and Critical Care Medicine, Ruijin Hospital, Shanghai Jiao Tong University School of Medicine, Shanghai, China; University of Pittsburgh School of Medicine

**Keywords:** *Klebsiella pneumoniae*, nonconjugative, virulence plasmid, resistance plasmid, IncN3 plasmid, mobilization

## Abstract

Klebsiella pneumoniae poses a critical challenge to clinical and public health. Along with conjugative plasmids, nonconjugative resistance or virulence plasmids associated with carbapenem-resistant K. pneumoniae (CRKP), hypervirulent K. pneumoniae (hvKP), and even carbapenem-resistant and hypervirulent K. pneumoniae (CR-hvKP) strains have been spreading globally. In this study, a clinical CRKP strain KP2648 was isolated, and the transferability of its plasmids was assessed using conjugation experiments. The transconjugants were characterized by polymerase chain reaction (PCR) detection, *Xba*I and S1-pulsed-field gel electrophoresis (PFGE), and/or whole-genome sequencing. Genetically modified IncN3 plasmids were employed to elucidate the self-transferability and the mobilization mechanisms. KP2648 has three natural plasmids: a nonconjugative IncFIB/IncHI3B virulence plasmid, a nonconjugative IncFII/IncR carbapenem-resistant plasmid, and a self-transferable IncN3 plasmid with a high conjugation frequency (7.54 ± 1.06) × 10^−1^. The IncN3 plasmid could mobilize the coexisting nonconjugative virulence/resistance plasmids either directly or by employing intermediate E. coli with two forms: a hybrid plasmid fused with IncN3 or a cotransfer with the helper plasmid, IncN3. Various mobile genetic elements, including IS*Kpn74*, IS*Kpn14*, IS*26*, IS*Shes11*, IS*Aba11*, and Tn*3,* are involved in the genetic transposition of diverse hybrid plasmids and the cotransfer process during the intra/interspecies transmission.

**IMPORTANCE** Nowadays, the underlying mobilization mechanism and evolutionary processes of nonconjugative virulence or resistance plasmids in Klebsiella pneumoniae remain poorly understood. Our study revealed the high conjugation ability of IncN3 plasmid isolated from carbapenem-resistant K. pneumoniae and confirmed its capability to mobilize the nonconjugative virulence or resistance plasmids. The self-transferable IncN3 plasmid could facilitate the transmission of pathogenicity and genetic evolution of carbapenem-resistant and hypervirulent K. pneumoniae, including hv-CRKP (virulence plasmid obtained by carbapenem-resistant K. pneumoniae) and CR-hvKP (resistance plasmid obtained by hypervirulent K. pneumoniae), warranting further monitoring.

## INTRODUCTION

Klebsiella pneumoniae has emerged as a common clinical and public health threat globally, with increasing prevalence among community-acquired and health care-associated infections (1). Numerous antimicrobial resistance (AMR) and virulence factors encoded by plasmids enhance the severity of K. pneumoniae infections and their propensity to cause invasive infections (2). Beyond carbapenem-resistant K. pneumoniae (CRKP) and hypervirulent K. pneumoniae (hvKP), the convergence of AMR and virulence genes in potentially newly emerging carbapenem-resistant and hypervirulent K. pneumoniae (CR-hvKP) has been documented as the cause of lethal outbreaks and extremely devastating clinical outcomes (3).

Mobile genetic elements (MGEs), such as plasmids and insertion sequence elements (ISs), play important roles in the multidimensional acquisition and dissemination of pathogenicity in *Enterobacteriaceae* (4). Among them, the horizontal gene transfer (HGT) of large conjugative plasmids encoding resistant genes, including the promiscuous incompatibility group F (IncF), IncN, and IncX, as well as other conjugative plasmids encoding virulence genes, mainly IncFIBK and IncHI1B, are the principal vehicles identified to be responsible for plasmid mobility, resulting in the development of CR-hvKP (5, 6). Plasmids lacking the conjugation machinery could be mobilized by other conjugative elements or by hybrid plasmid fusion through homologous recombination or intermolecular transposition (7, 8).

Classic pLVPK-like virulence plasmids are generally regarded as nonconjugative because they lack the *tra* gene cluster coding for a conjugation apparatus, which has likely limited their transmission, historically (9). However, recent studies have demonstrated that virulence genes or plasmids could be mobilized to access new hosts with the help of other integrative conjugative elements or conjugative resistance plasmids (10). Yang et al. reported the integration of a 100 kB fragment of pLVPK into a conjugative IncFIB plasmid in a clinical K. variicola strain and confirmed its self-transferability (11). Xie et al. discovered that an IncFIA plasmid could be fused to a hypervirulence-encoding plasmid to form a hybrid conjugative virulence plasmid via homologous recombination (12). We also previously reported the mobilization process of a nonconjugative virulence plasmid in the K. pneumoniae strain RJF293, which was transferred from an hvKP to a CRKP strain with the help of conjugative IncF plasmids via various modes (13). However, reports of mobilizations of pLVPK-like virulence plasmids or genes have predominantly focused on the IncF plasmids; thus, the mobilization phenomenon and the specific mechanism in other types of conjugative plasmids were rarely reported and await further study (11, 13).

Moreover, resistance to carbapenem is normally mediated by the production of carbapenemase, predominantly the K. pneumoniae carbapenemase (KPC) gene, which is typically plasmid-borne (14). Different *bla*_KPC_ isoforms are associated with different MGEs and are located in different *bla*_KPC_-harboring plasmids; thus, the horizontal transfer of different conjugative plasmids can explain the rapid dissemination of *bla*_KPC_ (10). However, several IncP- and IncQ-type plasmids lack *tra* genes and are nonconjugative. Truncation in the conjugation locus may also lead to the nontransferability of plasmids, as in the cases of the increasingly reported *bla*_KPC-2_-producing and nontransferred pKPC-LK30/pHN7A8-like plasmid in China. However, the underlying mechanisms contributing to the wide spread of the plasmids remain poorly understood (10, 15, 16).

Therefore, comprehensive genetic analysis is needed to explore the underlying transmission mechanisms of the nonconjugative virulence/resistance plasmids in *Enterobacteriaceae* (10). To this end, our study aimed to identify the presence of conjugative and nonconjugative plasmids in clinical K. pneumoniae isolates and to characterize their transmission ability, evolutionary process, and mobilization mechanisms. An intact IncN3 conjugative helper plasmid is reported, and mobilization mechanisms are explored using genetically modified plasmids. These findings can provide mechanistic insights, highlight the current threats of increased transmission efficiency, and broaden the host spectrum of Gram-negative bacteria via the rapid transmission of nonconjugative virulence/resistance plasmids.

## RESULTS

### Phenotypic and genomic characteristics of KP2648.

KP2648 was recovered from the anal swab of a 38-year-old male patient diagnosed with acute respiratory failure and cutaneous T-cell lymphoma at the intensive care unit (ICU) of Ruijin Hospital in 2019. He received methotrexate and prednisone, and he was immunocompromised throughout his hospitalization. Staphylococcus aureus, Enterococcus faecalis, carbapenem-susceptible K. pneumoniae, and E. coli were successively isolated from the skin wounds of the patient. CRKP strain KP2648 colonized the intestinal tract without causing infection manifestations. The patient showed typical symptoms of skin infections and was empirically treated with meropenem, vancomycin, fluconazole, and ganciclovir. His condition improved, and he was eventually discharged. KP2648 produced a negative string test and showed high resistance to a wide range of β-lactam antibiotics and carbapenem while remaining susceptible to colistin and tigecycline (Table 1).

**TABLE 1:** Phenotypic and genotypic characteristics of K. pneumoniae strain KP2648, E. coli C600, J53, and their corresponding transconjugants^*a*^

Strain	Species	MIC [μg mL^−1^]									*rmpA*	*bla*_KPC_	Conjugation efficiency
		CAZ	ATM	IPM	MEM	ETP	AMK	TIG	CST	CTX			
KP2648	K. pneumoniae	128	>128	32	128	>128	1	0.5	≤0.5	>128	+	+	
C600	E. coli	0.25	0.5	0.25	≤0.125	0.25	1	0.25	≤0.5	≤0.125	−	−	
EC2648-Vir1	E. coli	0.25	0.5	0.25	≤0.125	0.25	1	0.25	≤0.5	≤0.125	+	−	(4.26 ± 2.85) × 10^−5^
EC2648-Vir2	E. coli	0.25	0.5	0.25	≤0.125	0.25	1	0.25	≤0.5	≤0.125	+	−	(4.26 ± 2.85) × 10^−5^
EC2648-R1	E. coli	64	>128	16	128	16	1	0.25	≤0.5	>128	−	+	(3.75 ± 2.02) × 10^−6^
EC2648-R1-1	E. coli	64	>128	16	128	16	1	0.25	≤0.5	>128	−	+	(3.75 ± 2.02) × 10^−6^
EC2648-R2	E. coli	64	>128	16	128	16	1	0.125	≤0.5	>128	−	+	(3.75 ± 2.02) × 10^−6^
J53	E. coli	0.25	0.5	0.5	0.5	0.5	1	0.25	≤0.5	≤0.125	−	−	
J-Vir1	E. coli	≤0.125	0.5	0.5	0.5	0.5	1	0.25	≤0.5	≤0.125	+	−	(9.09 ± 4.64) × 10^−2^
J-Vir2	E. coli	0.25	0.5	≤0.25	≤0.25	0.5	1	0.25	≤0.5	≤0.125	+	−	(1.06 ± 0.46) × 10^−3^
J-R1	E. coli	64	>128	32	128	16	1	0.25	≤0.5	>128	−	+	(3.77 ± 1.66) × 10^−3^
J-R2	E. coli	64	>128	32	128	16	1	0.25	≤0.5	>128	−	+	(4.20 ± 1.44) × 10^−6^

a KP, *K. pneumoniae;* MICs, minimum inhibitory concentrations; CAZ, Ceftazidime; ATM, Aztreonam; IPM, Imipenem; MEM, Meropenem; ETP, Ertapenem; AMK, Amikacin; TIG, Tigecycline; CST, Colistin; CTX, Cefotaxime.

**FIG 1:**
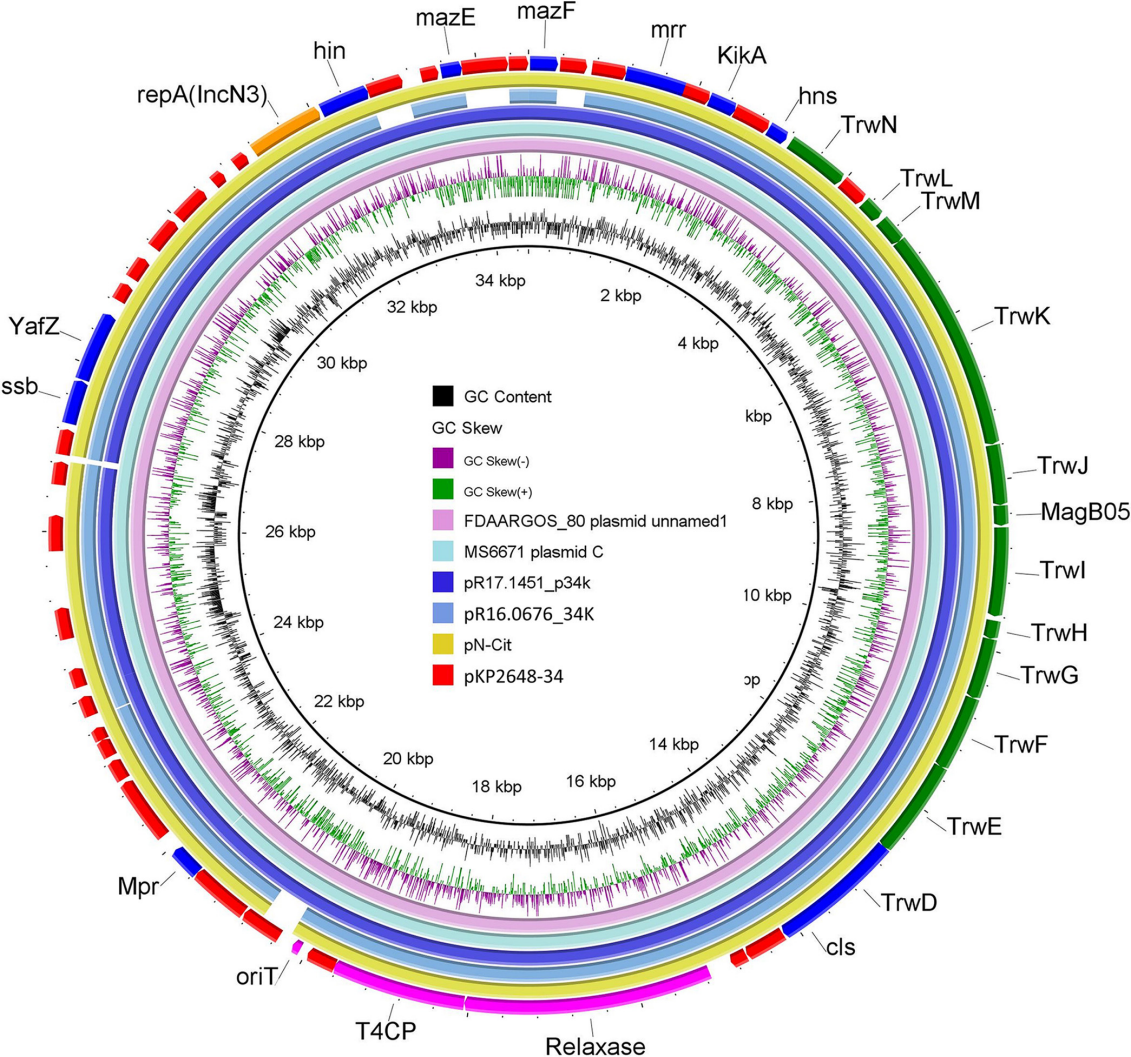
Sequence map of pKP2648-34 compared with other similar plasmids from GenBank. FDAARGOS_80 from P. mirabilis (accession number: NZ_CP026060), Plasmid_C_Kpneumoniae_MS6671 from K. pneumoniae (accession number: LN824136), pR17.1451_p34k from Salmonella (accession number: CP063297), pR16.0676_34k from Salmonella (accession number: NZ_CP029801), and pN-Cit from C. freundii (accession number: JQ996149).

Polymerase chain reaction (PCR) and whole-genome sequencing (WGS) showed that KP2648 belongs to ST11 and serotype K64. It has three natural plasmids, pKP2648-Vir (216 Kb), pKP2648-KPC (102 Kb), and pKP2648-34 (34 Kb). The IncFIB/IncHI3B plasmid pKP2648-Vir encoded the known virulence factors, such as the aerobactin siderophore, the regulator of mucoid phenotype A (*rmpA*), and the putative metabolite transporter (*peg344*). These virulence factor genes are usually co-localized on a large virulence plasmid and serve as reliable identifiers for hypervirulence (17). It also harbored the following ISs: IS*kpn74*, IS*1* family, IS*5075*, IS*630* family, IS*903B*, and others. The genomic sequence of pKP2648-Vir showed 91% coverage and 99.95% identity with that of the classical nonconjugative virulence plasmid pLVPK (9), and there was no *iro* deletion or *rmpA2* mutation (Fig. S1). The IncFII/IncR plasmid pKP2648-KPC contained several AMR genes, such as *bla*_KPC-2_, *bla*_CTX-M-65_, and *bla*_SHV-1_. Tn*3* and ISs (IS*kpn14*, IS*903B*, IS*5075*, IS*kpn27*, IS*26*, and IS*Kpn28*) were dispersed throughout the plasmid (Fig. S2). Sequence alignment showed that pKP2648-KPC is a pKPC-LK30/pHN7A8 plasmid that evolved from several recombination events of the nonconjugative *bla*_KPC-2_-carrying plasmid pKPC-LK30 and the conjugative *bla*_fosA3_-carrying plasmid pHN7A8. Notably, a part of the *tra* gene cluster of pHN7A8 (including *TrbC*, *TraU*, *TraW*, *TraC*, *TraV*, *TraB*, and *TraK*) was lost during the recombination (Fig. S3). Thus, the pKPC-LK30 and pHN7A8 hybrid plasmid pKP2648-KPC was presumed to be nonconjugative, which was confirmed by a conjugation assay. Such nonconjugative pKPC-LK30/pHN7A8-like plasmids were also reported in other K. pneumoniae CG258 strains (15, 16). The IncN3 plasmid pKP2648-34 did not contain any resistance/virulence genes; however, it did harbor a complete conjugative transfer-related module, including an *oriT* region, a relaxase of the MOBF family, a type IV coupling protein (T4CP) of the TraD family, and a *trw* gene cluster that coded for the IVB type T4SS. A BLASTN search showed that this IncN3 plasmid exhibited high sequence similarity (greater than 98% identity and coverage) to plasmid FDAARGOS_80 from Proteus mirabilis (accession: NZ_CP026060), Plasmid_C_Kpneumoniae_MS6671 from K. pneumoniae (LN824136), pR17.1451_p34k from Salmonella (CP063297), pR16.0676_34k from Salmonella (NZ_CP029801), and pN-Cit from Citrobacter freundii (JQ996149) (Fig. 1).

### Transferability of the nonconjugative virulence or resistance plasmids from KP2648 to E. coli.

**FIG 2:**
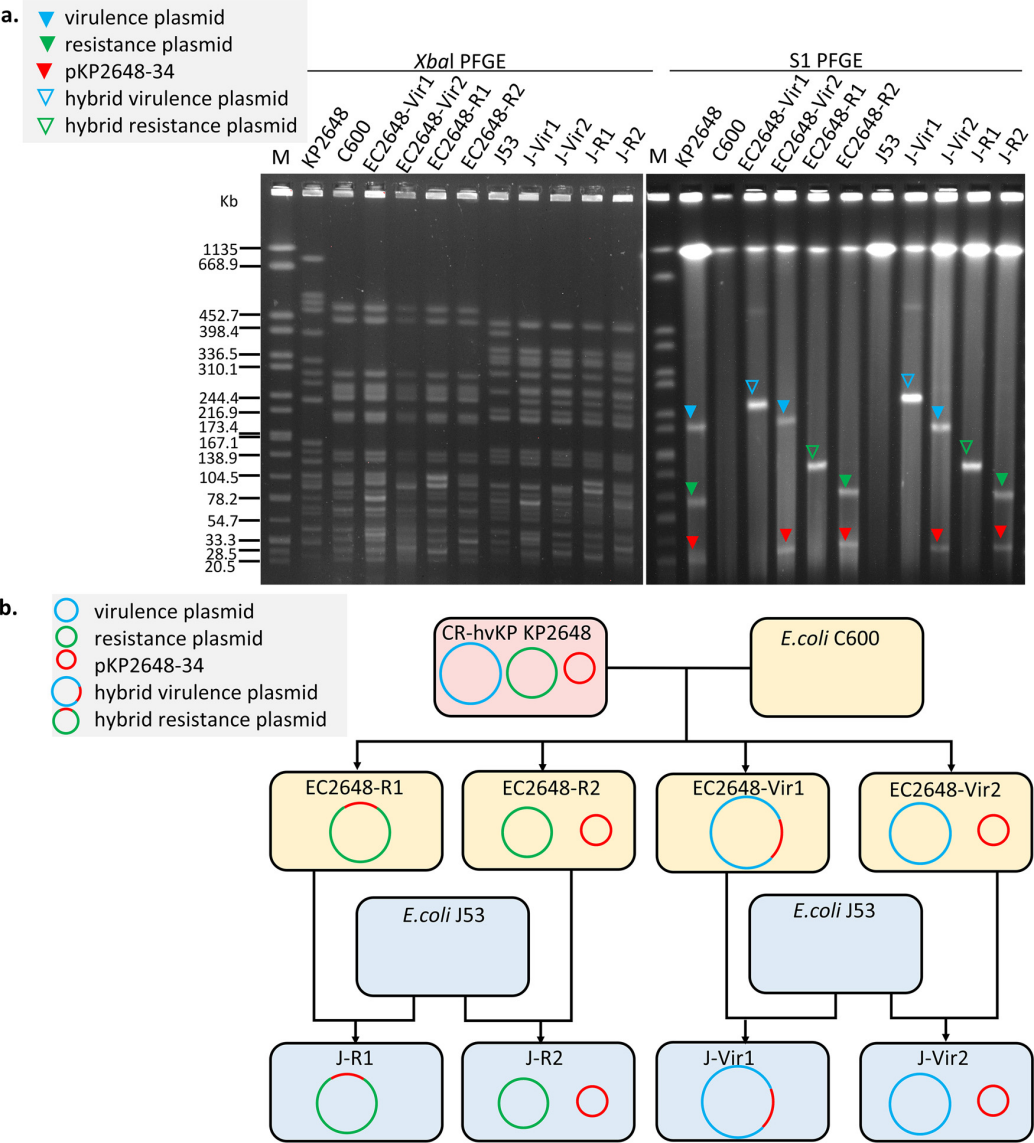
Transfer of the nonconjugative virulence or resistance plasmids of KP2648 to E. coli. (a) *Xba*I and S1-PFGE of K. pneumoniae KP2648, E. coli C600, E. coli J53, and their corresponding transconjugants. EC2648-Vir1 and EC2648-Vir2 were detected by conjugation to select the virulence plasmid, whereas EC2648-R1 and EC2648-R2 were obtained by selecting resistance transconjugants. These transconjugant plasmids could be transferred to J53, represented as J-Vir1, J-Vir-2, J-R1, and J-R2. Differently colored triangles denote different plasmids; subsequent WGS confirmed that plasmids represented by the same triangles had similar backbones with or without additional ISs. (b) Schematic representation of the conjugation assays of KP2648 to E. coli. Rounded rectangles of the same color represent the same strains. Blue, green, and red circles denote pKP2648-Vir or its derivatives, pKP2648-KPC or its derivatives, and pKP2648-34, respectively.

Conjugation experiments were performed to evaluate the transferability of the nonconjugative virulence or resistance plasmids from KP2648. pKP2648-Vir was transferred from KP2648 to E. coli C600 (Fig. 2). Two different patterns of transconjugants, EC2648-Vir1 and EC2648-Vir2, were observed with a conjugation efficiency of 10^−5^ during the selection of the virulence plasmid. The two virulence transconjugants were susceptible to carbapenem, and PCR results were *rmpA*-positive and *bla*_KPC-2_-negative (Table 1). S1 nuclease pulsed-field gel electrophoresis (S1-PFGE) showed that EC2648-Vir1 contained a hybrid virulence plasmid (represented as pVir-fusion), which was speculated to have been derived from the fusion of pKP2648-Vir and pKP2648-34. EC2648-Vir2 contained two separate plasmids with sizes similar to those of pKP2648-Vir and KP2648-34, respectively. The virulence plasmids from both EC2648-Vir1 and EC2648-Vir2 were stable after serial batch culturing for 30 passages. In addition, both of these transconjugants could subsequently transfer their virulence plasmids to J53 in the same structures with higher frequencies of 10^−2^ and 10^−3^, respectively. We further observed that the transfer derivative of pKP2648-Vir in EC2648-Vir2 was accompanied by pKP2648-34 in conjugation experiments.

The resistance plasmid pKP2648-KPC, which does not carry the intact *tra* gene cluster, was transferred to C600 (Fig. 2). Three different types of resistance transconjugants (EC2648-R1, EC2648-R1-1, and EC2648-R2) were selected with a conjugation efficiency of 10^−6^ (Table 1). EC2648-R1 and EC2648-R1-1 harbored similar hybrid resistance plasmids, which were speculated to be the fusion of pKP2648-KPC and pKP2648-34 (pKPC-fusion and pKPC-fusion-1). EC2648-R2 contained two separate plasmids with sizes similar to those of pKP2648-KPC and KP2648-34, respectively. Also, the resistance plasmids from EC2648-R1 and EC2648-R2 were stable. Interestingly, pKPC-fusion from EC2648-R1 could be subsequently transferred to J53, whereas pKPC-fusion-1 could not be transferred at all. Similarly, the transfer of the resistance plasmid in EC2648-R2 was accompanied by pKP2648-34.

However, we did not observe the simultaneous dissemination of pKP2648-Vir and pKP2648-KPC in a single event using the selection with the meropenem, potassium tellurite (K_2_TeO_3_), and rifampicin screening agar.

### Transmission of the nonconjugative virulence or resistance plasmids to CRKP or hvKP, respectively.

**FIG 3:**
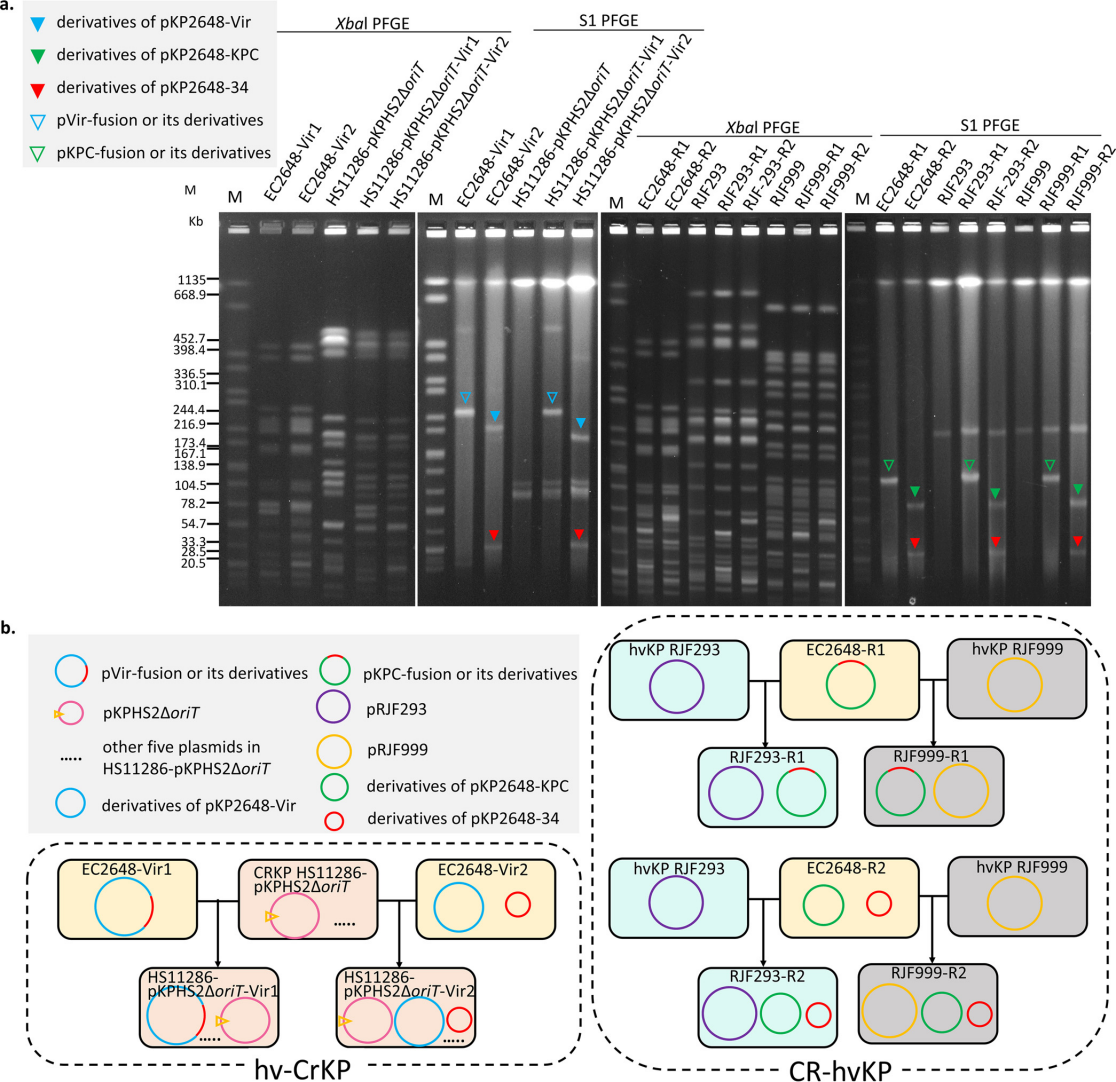
Transmission of the nonconjugative virulence or resistance plasmids to CRKP or hvKP, respectively. (a) *Xba*I and S1-PFGE of EC2648-R1, EC2648-R2, EC2648-Vir1, EC26480Vir2, HS11286-pKPHS2Δ*oriT*, RJF293, RJF999, and their corresponding transconjugants. The meaning of different colored triangles is consistent with that in Fig. 1. (b) Schematic of the process for the formation of hv-CRKP and CR-hvKP. The open yellow triangle represents the deletion of the *oriT* region.

To test whether the virulence or resistance plasmids of the E. coli transconjugants obtained above could be transferred to K. pneumoniae strains, one CRKP strain (HS11286-pKPHS2*ΔoriT*) and two hvKP strains (RJF293 and RJF999) were employed as individual recipients (Fig. 3 and Table S1). EC2648-Vir1 and EC2648-Vir2 could be successfully transferred to HS11286-pKPHS2Δ*oriT*, and EC2648-R1 and EC2648-R2 could also be transferred to RJF293 and RJF999. Fig. 3b outlines the formation process of hv-CRKP and CR-hvKP by an intermediate E. coli.

To investigate whether these plasmids could be directly transferred from KP2648 to other CRKP or hvKP, we observed that the pKP2648-Vir was successfully transferred from KP2648 to HS11286-pKPHS2Δ*oriT* in a fusion form with a conjugation frequency of (7.24 ± 0.82) × 10^−7^ (Table S1). However, the transfer of pKP2648-KPC was not observed in the direct conjugation between KP2648 and RJF293H. To avoid the inhibitory effects of hyperviscosity, the *rmpA*-deficient RJF293ZH was also used as the recipient strain, but pKP2648-KPC was still not observed.

### Genetic basis of hybrid plasmid generation.

**FIG 4:**
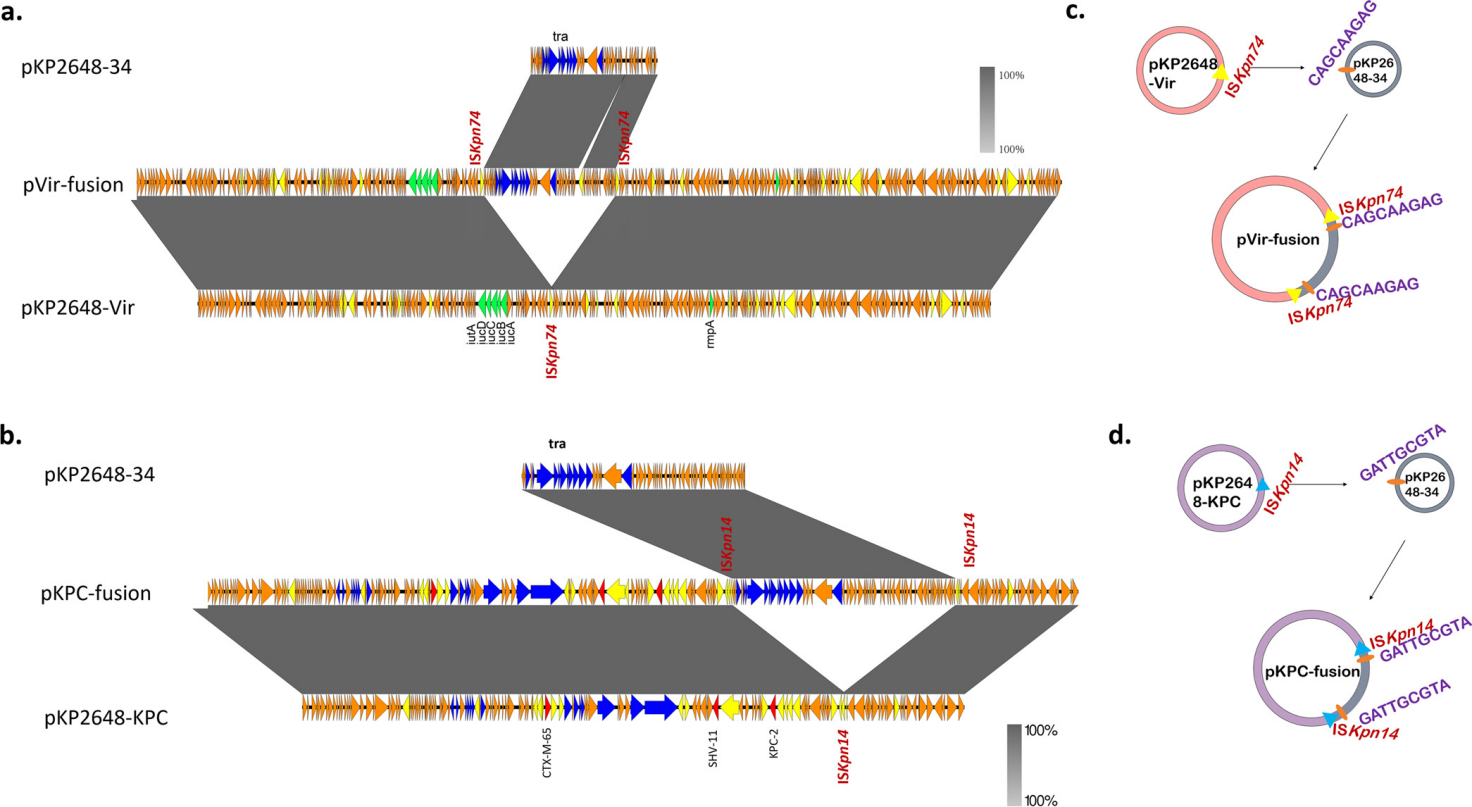
Genetic structures of the conjugative hybrid virulence plasmid pVir-fusion and the hybrid resistance plasmid pKPC-fusion. Alignment of pVir-fusion (a) and pKPC-fusion (b) with parental plasmids from KP2648. Linear comparison figures were created in Easyfig (http://easyfig.sourceforge.net/). Blue, green, and yellow arrows indicate *tra* genes, virulence genes, and ISs, respectively. (c) Proposed mechanisms of plasmid fusion in pVir-fusion: IS*kpn74* on pKP2648-Vir attacked a 9 bp target site duplication (CAGCAAGAG) of pKP2648-34, leading to the formation of pVir-fusion through a replicative transposition mechanism. (d) Proposed mechanisms of plasmid fusion in pKPC-fusion: IS*Kpn14* located in plasmid pKP2648-KPC may attack a 9 bp target site duplication (GATTGCGTA) of pKP2648-34, leading to the formation of pKPC-fusion.

To explore the possible mobilization mechanism of the nonconjugative pKP2648-Vir and pKP2648-KPC plasmids, five virulence- and resistance-related transconjugants (EC2648-Vir1, EC2648-Vir2, EC2648-R1, EC2648-R1-1, and EC2648-R2) were sequenced. Genomic sequence alignments revealed that the hybrid plasmids represented various fusion products of the nonconjugative virulence/resistance plasmids with the conjugative IncN3 plasmid pKP2648-34. Notably, IS*Kpn74* elements located in pKP2648-Vir presumably attacked a 9 bp target site duplication (CAGCAAGAG) of pKP2648-34 through replicative transposition, resulting in the formation of a hybrid pVir-fusion in EC2648-Vir1 (Fig. 4a and c). Similarly, IS*Kpn14* located in pKP2648-KPC might attack a 9 bp target site duplication (GATTGCGTA) of pKP2648-34, leading to the formation of pKPC-fusion (Fig. 4b and d). However, we did not observe the transfer of pKPC-fusion-1 in EC2648-R1-1. It might be due to the interruption of the *trwJ* gene that codes for the T4SS component by an IS*26* insertion (Fig. S4).

In addition, we noticed that the Tn*3* transposon was only present in pKP2648-KPC among the plasmids of KP2648, though it was added to pKP2648-VirR and to pKP2648-34R in the transconjugants EC2648-Vir2 and EC2648-R2. This observation suggests that the replicative transposition of Tn*3* might contribute to the dissemination of these virulence and resistance plasmids (Fig. S5).

### Mobilization of the nonconjugative virulence/resistance plasmids by the IncN3 plasmid.

**FIG 5:**
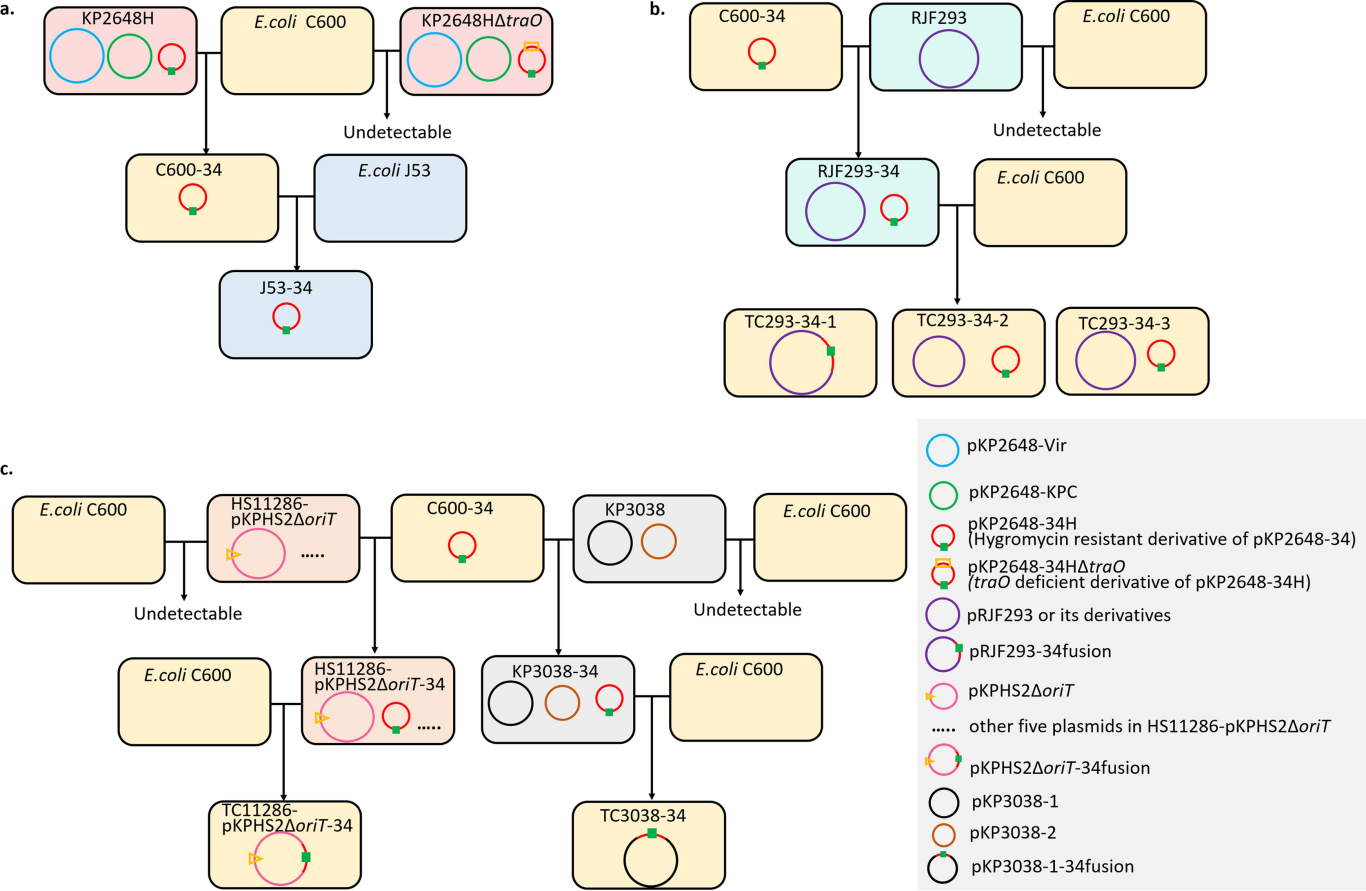
Mobilization of the nonconjugative pLVPK-like virulence plasmid and the nonconjugative resistance plasmid with the help of pKP2648-34. (a) Mutant pKP2648-34H could transfer to E. coli C600 and then E. coli J53 independently; *traO*-deficient pKP2648-34HΔ*traO* failed to transfer and detect in the conjugation between KP2648HΔ*traO* and E. coli C600. (b) pKP2648-34H could transfer to RJF293, enabling the transmission of a nonconjugative virulence plasmid. (c) pKP2648-34H could transfer to KP3038 and HS11286-pKPHS2Δ*oriT* and helped the transmission of a nonconjugative resistance plasmid. The green square denotes the hygromycin resistance gene on the IncN3 plasmid.

From the above-described experiments, we speculate that the IncN3 plasmid has a strong conjugation ability and plays a critical role in mobilizing nonconjugative virulence or resistance plasmids. To identify the transfer ability of the IncN3 plasmid, the hygromycin resistance gene was inserted into the IncN3 plasmid, named pKP2648-34H, and the modified KP2648 strain was named KP2648H, accordingly. Modified pKP2648-34H and KP2648H strains were constructed for further conjugation screening (Fig. 5). Finally, pKP2648-34H could be transferred to C600 independently with a high conjugation frequency of (7.54 ± 1.06) × 10^−1^. Furthermore, the self-transferable pKP2648-34H could also be transferred to E. coli J53, RJF293, KP3038, and HS11286-pKPHS2Δ*oriT* with a high conjugation efficiency of 10^−1^ (Table S1).

In the conjugation between C600 and RJF293-34, various modes of pRJF293 transconjugants were observed (Fig. S6): (i) pRJF293 fused with pKP2648-34H in the transconjugant TC293-34-1; (ii) pRJF293 cotransfer with pKP2648-34H in TC293-34-2; and (iii) TC293-34-3, a shortened form of pRJF293 transferred with pKP2648-34H. Similarly, pKP2648-34H also contributes to the mobilization of the nonconjugative KPC plasmids in HS11286-pKPHS2Δ*oriT*-34 and KP3038-34, likely via a mechanism of plasmid fusion (Fig. S6). PCR and sequencing analysis showed that IS*Shes11* and IS*Aba11* participate in the fusion formation of TC293-34-1 and TC11286-pKPHS2Δ*oriT*-34. We then knocked out the *traO* gene of pKP2648-34H, which encodes the VirB9 protein that is responsible for the core complex of T4SS. The *traO*-deficient pKP2648-34HΔ*traO* failed to transfer during the conjugation between KP2648HΔ*traO* and C600. Moreover, neither the virulence plasmids nor the resistance plasmids were transferred in the conjugation. Altogether, these results showed that the self-transferable IncN3 plasmid could mobilize the nonconjugative virulence or KPC resistance plasmids either via cotransfer or via hybrid formation mediated by various MGEs.

In addition, we designed the PCR primers specific to the conserved region of the IncN3 plasmid to investigate if it is commonly present in clinical K. pneumoniae. A total of 509 clinical K. pneumoniae isolates were screened, and 1.57% (8/509) of the isolates contained the IncN3 plasmid, including three CRKP strains, five CSKP strains, and one of CSKP strains is hvKP. Although the IncN3 plasmids were detected sporadically, it was not surprising that most strains (6/8) were observed among the critically ill patients in ICUs.

## DISCUSSION

CRKP has been registered in the critical priority tier by the World Health Organization and has become a major menace of a public health concern (18). Moreover, CR-hvKP has recently become even more prevalent than previously assumed. Several strains are emerging with different carbapenemase-producing genes, including *bla*_KPC_, *bla*_NDM_, and *bla*_OXA_, posing a great challenge to infection control (3, 19, 20). The gut microbiome is the major reservoir and hot spot for exchanging virulence and carbapenemase-encoding genes during in-host evolution (21). Gastrointestinal colonization of CRKP or CR-hvKP, particularly in ICU wards, may lead to subsequent infections in at-risk patients, potentially playing a role in clone or plasmid outbreaks (22, 23). Therefore, although collected from a stool sample, KP2648 should not be neglected, as it was isolated from an immunodeficient patient in an ICU. Thus, active culture surveillance and innovative strategies are urgently needed to curtail the further widespread transmission of CR-hvKP in nosocomial settings in China.

In addition to chromosomal mutations, the acquisition of pathogenic genetic element resistance and virulence phenotypes is mainly driven by plasmids (4). According to their mobility ability, plasmids can be assigned to three groups: conjugative, mobilizable, and nonmobilizable (4). Most notoriously, conjugative plasmids are especially relevant and indispensable for pathogenic gene transmission in different bacterial species (24). However, it should be noted that nonconjugative resistance or virulence plasmids associated with CRKP or even CR-hvKP exhibit global dissemination. However, the underlying mobilization mechanisms and evolutionary processes of these nonconjugative plasmids remain poorly understood.

The recognition of conjugative components or conjugative helper plasmids is essential in predicting the likelihood of the dissemination of nonconjugative plasmids (10). Previous studies on conjugative helper plasmids mainly focused on resistance plasmids. For example, the Incl1 conjugative helper plasmid has been shown to facilitate the transmission of the nonconjugative ciprofloxacin resistance plasmid in Salmonella (25). To the best of our knowledge, our study is the first to demonstrate that the IncN3 plasmid plays a unique functional role as a conjugative helper plasmid and that it could transfer either the nonconjugative pLVPK-like virulence plasmid or the nonconjugative pKPC-LK30/pHN7A8-like resistance plasmid during the evolution of CR-hvKP. IncN plasmids have a broad host range. The IncN3 plasmid carries a complete conjugative transfer module. It is confirmed as self-transmissible with a high conjugation efficiency of (7.54 ± 1.06) × 10^−1^ and is stably inherited in the transconjugants (26). A BLASTN search showed that the IncN3 plasmid is also present in other *Enterobacteriaceae* species, including Proteus mirabilis, Salmonella spp., Enterobacter hormaechei, Shigella dysenteriae, and Citrobacter freundii. The IncN3 plasmid was first reported and designated pN-Cit in C. freundii. In this report, pN-Cit was predicted to promote the mobilization of the nonconjugative pT-OXA-181 plasmid (27). It was also reported to probably facilitate the mobilization of the nonconjugative resistance plasmid pR16.0676_90k in Salmonella (28). In addition, a mosaic *bla*_KPC-2_-harboring plasmid might have emerged via the incorporation of Tn*4401* by an IncN1-type replicon and an IncN3-type *tra* system (29). Collectively, these findings indicate that the IncN3 plasmid might have a powerful ability to transfer various nonconjugative plasmids or mobile elements across different species in *Enterobacteriaceae*. Furthermore, our study demonstrated that IncN3 plasmids might have greatly accelerated the formation of hv-CRKP and CR-hvKP by mobilizing nonconjugative plasmids either directly or indirectly. In addition, the IncN3 plasmid and the novel transconjugant plasmids might transfer freely in the gut microbiota of patients, thereby drastically broadening the transmission efficiency of pathogenicity genes and expanding the host spectrum. Thus, exploiting the conjugative delivery function of the IncN3 plasmid and interrupting plasmid-mediated transmission are warranted in further microbiome modification research (30).

Plasmids often contain multiple MGEs that enable them to undergo frequent genetic transposition, resulting in plasmid fusion or rearrangement, which leads to a better adaptation to the bacterial host (4). ISs are prevalent and dynamically distributed at a higher abundance on plasmids. ISs could capture and attack the hot spot in the conjugative helper plasmid to trigger homologous recombination and intermolecular transposition (24). It is reported that ISs participate in 63.2% of the transferred AMR genes between plasmids and chromosomes (24). For instance, IS*26* and IS*1216E* (IS*6* family) could mediate plasmid fusion and transmit resistance-encoding genes (25, 31). Besides, replicative transpositions were reported in other presumed MGEs, such as Tn*4401* (Tn*3* family), IS*903B* (IS*5* family), and IS*Kpn14* (IS*1* family) (32, 33). In our study, a diverse range of MGEs, including IS*Kpn74*, IS*Kpn14*, IS*26*, IS*Shes11*, IS*Aba11*, and Tn*3*, allowed for the identification of the hot spot of the IncN3 plasmid. These MGEs foster opportunities for plasmid fusion and transposition events across different genetic backgrounds. The continuous emergence and diversity of the IncN3 plasmid-mediated mobilization mechanism in *Enterobacteriaceae* should be further studied.

In summary, we have provided a report on the emergence of a self-transferable IncN3 plasmid with a broad host range that serves as a conjugative helper plasmid in K. pneumonia. It could directly mobilize the nontransmissible pLVPK-like or pKPC-LK30/pHN7A8-like plasmids or employ intermediate E. coli in the evolutionary processes of CR-hvKP. Two transfer forms were conserved: a hybrid plasmid fusion with the IncN3 plasmid through a replicative transposition mechanism and a simultaneous but separate transfer with the IncN3 plasmid. We further confirmed the detailed mobilization mechanisms of the IncN3 plasmid in different types of K. pneumoniae and outlined the genetic evolution processes of hv-CRKP and CR-hvKP. The high conjugation ability to a new host and the stable inheritance of IncN3 would significantly speed up the transmission of pathogenicity in *Enterobacteriaceae*, warranting continuous surveillance.

## MATERIALS AND METHODS

### Ethics approval.

The K. pneumoniae strain KP2648 and other clinical isolates were collected from patients admitted to the Ruijin Hospital during routine clinical treatments. All personally identifiable information was removed before analysis. The study protocol was approved by the Ethics Committee of Ruijin Hospital, School of Medicine, Shanghai Jiao Tong University (RJ2019NO1-3). The requirement for informed consent was waived.

### Bacterial strains and plasmids.

The bacterial strains and plasmids used are listed in Table S2. KP2648 was collected from a stool swab and confirmed as K. pneumoniae by matrix-assisted laser desorption ionization-time of flight mass spectrometry (bioMérieux, Marcy-l’Étoile, France). For the different conjugation assays, rifampicin-resistant E. coli C600, sodium azide-resistant E. coli J53, K. pneumoniae strains, including RJF293, RJF999, HS11286, its derivative HS11286-pKPHS2Δ*oriT* (*bla*_KPC-2_ encoding plasmid pKPHS2 cannot be transferred due to the deletion of *oriT*), and clinical isolate KP3038, were employed as the recipient strains (13). hvKP strain RJF293 (NCBI accession number: PRJNA307277) (34) had a capsular serotype K2 and ST374, whereas RJF999 (PRJNA307276) was of serotype K1 and ST23. RJF293H is a derivative of RJF293 with a chromosomal insertion of the hygromycin resistance gene (*hph*), while RJF293ZH is a derivative of RJF293H with *rmpA* gene deletion. The ST11 CRKP strain HS11286 (PRJNA78789) was isolated from the human sputum (35). The clinical CRKP strain KP3038 was isolated from an anal swab specimen that was positive for ST11 and *bla*_KPC-2_; however, the transferability of the *bla*_KPC-2_ gene of KP3038 cannot be detected by a conventional conjugation assay. In addition, 509 clinical K. pneumoniae isolates were collected as part of the routine clinical work with hospitalized patients from July to December 2020.

### Multilocus sequence typing (MLST), antimicrobial susceptibility testing (AST), and string testing.

Seven housekeeping genes for K. pneumoniae were amplified, sequenced, and analyzed according to the MLST database (36). ASTs of the strains were initially performed using the VITEK2 compact system (bioMérieux, Marcy-l’Étoile, France) and were then determined by the broth microdilution method. Results were interpreted according to the Clinical and Laboratory Standards Institute guidelines (M100-ED30), except for tigecycline (37). In addition, the hypermucoviscosity phenotype was determined by a string test, conducted as described in our previous work (34).

### PCR.

The oligonucleotide primers used are listed in Table S3. PCR was conducted to detect key carbapenem-resistance genes, virulence genes, and specific and important backbone genes in plasmids pKP2648-Vir, pKP2648-KPC, and pKP2648-34. PCR results were confirmed by direct Sanger sequencing.

### Plasmid conjugation assay and plasmid stability.

The transferability of pKP2648-Vir, pKP2648-KPC, pKP2648-34H, and its derivative pKP2648-34HΔ*traO* was assessed by conjugation experiments (11). We further tested the conjugative mobilization function of the self-transferable IncN3 plasmid. Donor and recipient isolates and antibiotics used for each pair of conjugation assays are listed in Table S4. Transconjugants were validated by AST, PCR, *Xba*I, S1 nuclease PFGE, and/or WGS (13). The conjugation frequency was calculated as the ratio of the number of transconjugants to recipient cells. Data were presented as mean ± standard deviation (SD) based on three independent experiments. Furthermore, serial culturing of the purified transconjugants EC2648-Vir1, EC2648-Vir2, EC2648-R1, and EC2648-R2 was carried out for 30 generations in lysogeny broth (LB) by transferring 100 μL of bacterial culture to 10 mL of fresh LB every 12 h. Plasmid stability was assessed by randomly selecting single bacterial colonies for antibiotic resistance verification and PCR detection of key resistance/virulence genes or specific backbone genes of the IncN3 plasmid.

### *Xba*I and S1 nuclease PFGE.

*Xba*I and S1-PFGE were performed to confirm the genetic relatedness and the acquisition of different plasmids by the recipient strains. Chromosomal and plasmid DNA of the strains were prepared in agarose blocks and digested with *Xba*I and S1 nuclease, respectively. DNA fragments were separated by PFGE on the CHEF Mapper XA system (Bio-Rad, Hercules, CA, USA) (13).

### WGS, assembly, and annotation.

The genomic DNA of KP2648 and five transconjugants (EC2648-Vir1, EC2648-Vir2, EC2648-R1, EC2648-R1-1, and EC2648-R2) were extracted and sequenced using a combination of the 150 bp paired-end Illumina NovaSeq 6000 platform and the PacBio RSII single-molecule long-read sequencing platform. Reads were reassembled using HGAP and the Canu 2.0 software package (38). The genomic data were annotated using Prokka 1.1.3 (39). Plasmid incompatible types were analyzed by PlasmidFinder 2.1 (40). MGEs, virulence, and antibiotic resistance determinants were predicted by VRProfile (41). oriTfinder was used to determine the conjugative transfer-related modules of the plasmids, including the relaxase gene, the type IV coupling protein (T4CP) gene, and the *tra* gene cluster associated with the type IV secretion system (T4SS) (42). Plasmid sequences were aligned using the BLAST Ring Image Generator and Easyfig (43, 44).

### Genetically modified IncN3 plasmid.

To verify the conjugation ability of the IncN3 plasmid, the hygromycin resistance gene *hph* was inserted into pKP2648-34. Thermo-sensitive helper plasmid pKOBEG carries the apramycin resistance gene and overexpresses the *exo*, *bet*, and *gam* genes of λ phage (13, 45). pKOBEG was electrically transferred into KP2648 competent cells, and positive cells were identified by apramycin. Positive cells were cultured in LB containing apramycin, and arabinose was added to induce the expression of the *Exo*/*BET*/*GAM* proteins for homologous recombination. Homologous recombinant fragments (upstream homologous arm-*hph*-downstream homologous arm) were prepared by gene splicing overlap extension-PCR and were electrically transferred into competent cells. The KP2648H strain with *hph* inserted was selected using a hygromycin-containing medium. The *traO* gene encodes the VirB9 protein, which is responsible for the core complex of the T4SS of the IncN3 plasmid (46). The *traO* gene was knocked out from the KP2648H isolate using similar methods. The PCR primers used for the construction of the modified IncN3 plasmids are listed in Table S3 (47).

### Data availability.

The genome sequences of K. pneumoniae KP2648, E. coli EC2648-Vir1, EC2648-Vir2, EC2648-R1, EC2648-R1-1, and EC2648-R2 have been deposited in the GenBank database under accession numbers CP072555-CP072558, CP083610-CP083611, CP083990-CP083992, CP084205-CP084206, CP084207-CP084208, and CP084209-CP084211, respectively.
