# Supplementary material for: Transmission of Nonconjugative Virulence or Resistance Plasmids Mediated by a Self-Transferable IncN3 Plasmid from Carbapenem-Resistant Klebsiella pneumoniae
Source: Microbiol Spectr. 2022 Jul 14;10(4):e01364-22. doi: 10.1128/spectrum.01364-22 (PMC9430514; doi:10.1128/spectrum.01364-22)
Supplement: Supplemental file 1 — Supplemental material. Download spectrum.01364-22-s0001.pdf, PDF file, 1.4 MB [file spectrum.01364-22-s0001.pdf]

Supplemental files

Figure S1. Schematic map of pKP2648-Vir compared with that of other virulence plasmids from GenBank.

Figure S2. Sequence map of pKP2648-KPC compared with that of other resistance plasmids from GenBank.

Figure S3. Structural features of pKP2648-KPC compared with those of pHN7A8 and pKPC-LK30.

Figure S4. Genetic structures of the non-conjugative hybrid resistance plasmid, pKPC-fusion-1.

Figure S5. Tn*3* transposon with the putative contributions to the dissemination of virulence and resistance plasmids in separate forms of transconjugants.

Figure S6. XbaI PFGE and S1-PFGE of modified KP2648H and a series of transconjugants.

Table S1. Phenotypic and genotypic characteristics of K. pneumoniae strain RJF293, RJF999, HS11286-pKPHS2 oriT KP3038 and their corresponding transconjugants.

Table S2. Strains and plasmids used in this study.

Table S3. Oligonucleotides used in this study.

Table S4. Antibiotics and the corresponding concentrations used for each conjugation pair.

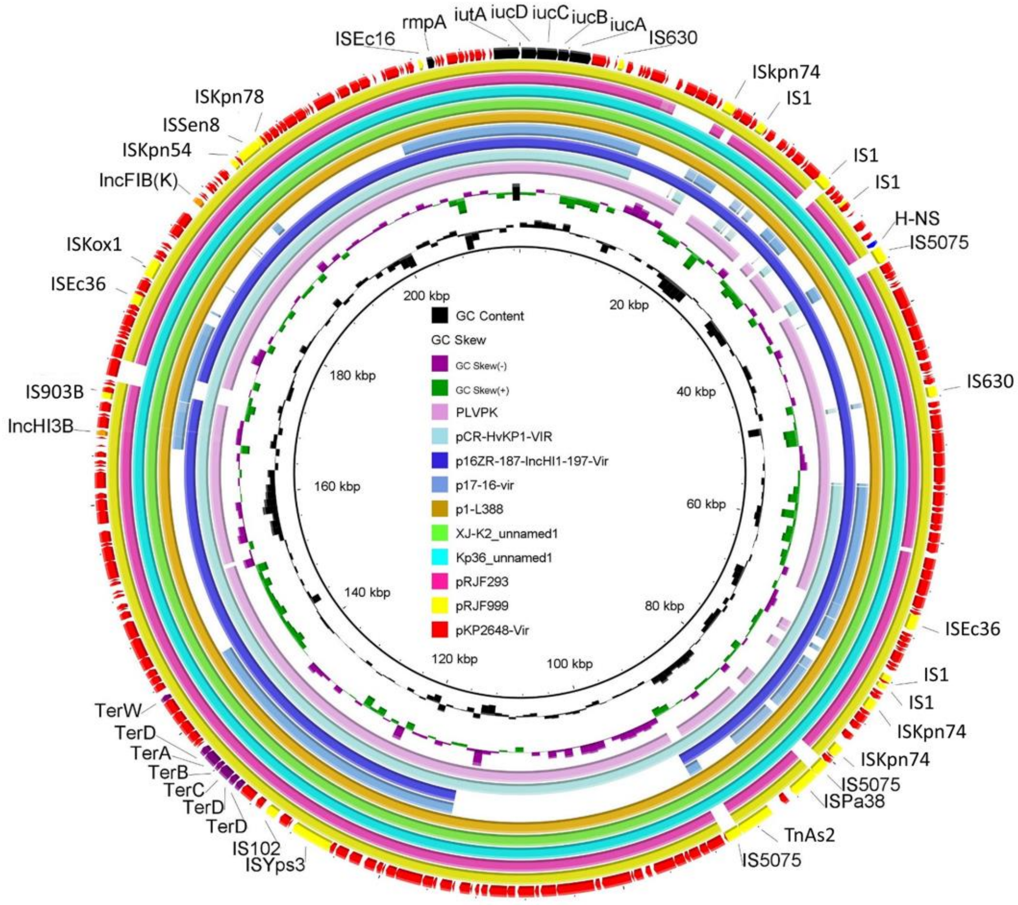

Figure S1. Schematic map of pKP2648-Vir compared with that of other virulence plasmids from GenBank. The circular map was created using BRIG (http://sourceforge.net/projects/brig/).

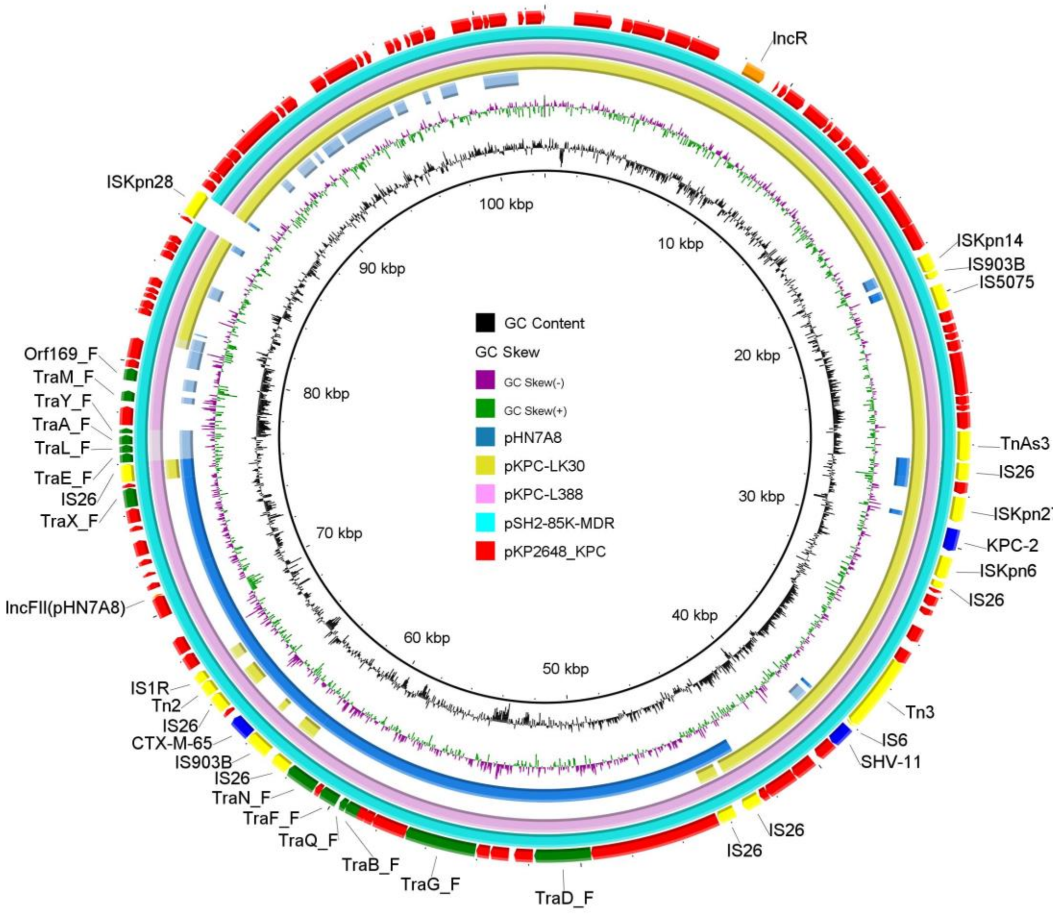

Figure S2. Sequence map of pKP2648-KPC compared with that of other resistance plasmids from GenBank.

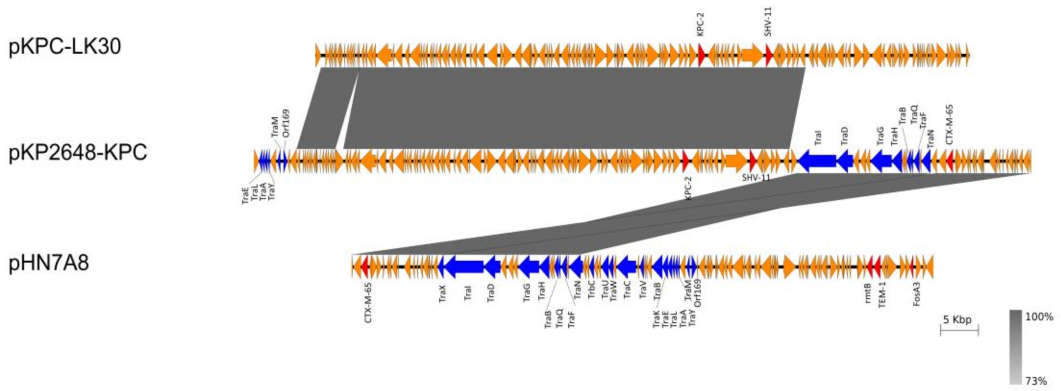

Figure S3. Structural features of pKP2648-KPC compared with those of pHN7A8 and pKPC-LK30. Blue and red arrows indicate *tra* and resistance genes, respectively.

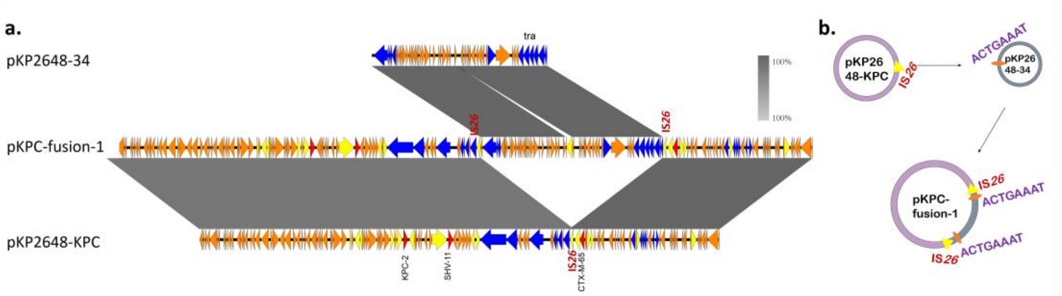

Figure S4. Genetic structures of the non-conjugative hybrid resistance plasmid, pKPC-fusion-1. (a) Alignment of the hybrid resistance plasmid, pKPC-fusion with parental plasmids from KP2648. (b) pKPC-fusion-1 in EC2648-R1-1 could not be transferred, probably because the hot spot (ACTGAAAT) in the *TrwJ* gene involved in the T4SS system of pKP2648-34 was interrupted by the IS*26*-mediated transposition process.

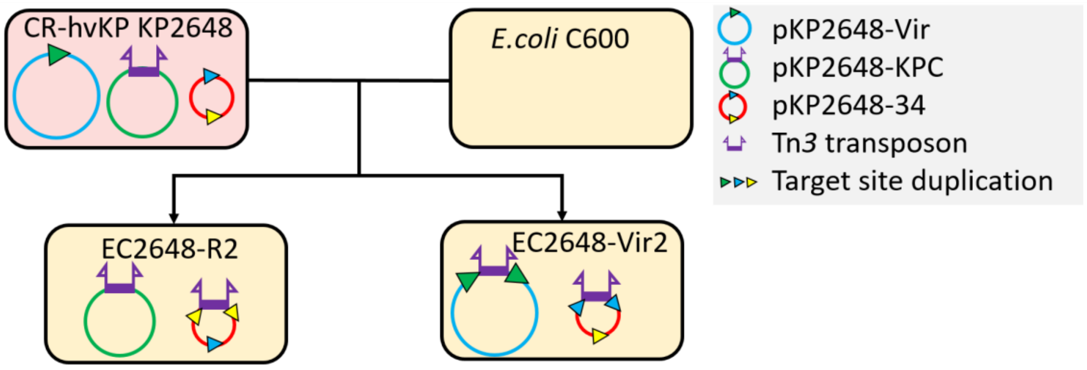

Figure S5. Tn3 transposon with the putative contributions to the dissemination of virulence and resistance plasmids in separate forms of transconjugants. The Tn*3* transposon is only harbored by pKP2648-KPC in KP2648 but is incorporated in separate plasmids, *i.e.*, pKP2648-VirR and pKP2648-34R in the transconjugants EC2648-Vir2 and EC2648-R2.

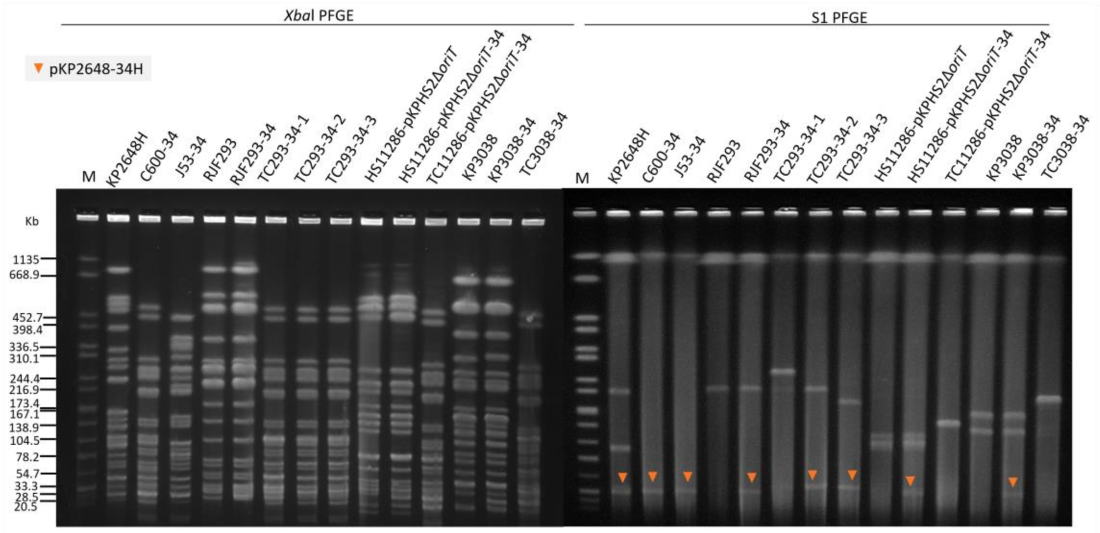

Figure S6. *Xba*I PFGE and S1-PFGE of modified KP2648H and a series of transconjugants. Full details of the strains are provided in Supplementary Table S1. Solid orange triangles denote pKP2638-34H.

Table S1. Phenotypic and genotypic characteristics of *K. pneumoniae* strain RJF293, RJF999, HS11286-pKPHS2Δ*oriT*， KP3038 and their corresponding transconjugants.

Strain ID	Species	MIC[ug ml-1]									*rmpA*	*bla*$_{KPC}$	Conjugation efficiency
		CAZ	ATM	IPM	MEM	ETP	AMK	TIG	CST	CTX			
RJF293	*K. pneumoniae*	<0.125	0.5	0.5	0.5	0.5	1	1	≤0.5	≤0.125	+	-	/
RJF293-R1	*K. pneumoniae*	32	>128	>128	64	64	1	1	≤0.5	>128	+	+	$(6.00±0.64)×10^{-5}$
RJF293-R2	*K. pneumoniae*	32	>128	>128	64	32	1	1	≤0.5	>128	+	+	$(1.20±0.65)×10^{-6}$
RJF999	*K. pneumoniae*	<0.125	0.5	0.5	0.5	0.5	1	1	≤0.5	≤0.125	+	-	/
RJF999-R1	*K. pneumoniae*	16	64	32	16	32	2	1	≤0.5	>128	+	+	$(2.85±1.96)×10^{-5}$
RJF999-R2	*K. pneumoniae*	16	64	32	16	32	1	1	≤0.5	>128	+	+	$(9.56±2.58)×10^{-8}$
HS11286	*K. pneumoniae*	32	64	32	32	64	64	1	≤0.5	>128	-	+	/
HS11286-pKPHS2Δ*oriT*	*K. pneumoniae*	32	64	32	32	64	64	1	≤0.5	>128	-	+	/
HS11286-pKPHS2 Δ *oriT*-Vir1	*K. pneumoniae*	64	64	32	32	64	64	1	≤0.5	>128	+	+	$(1.38±0.89)×10^{-4}$
HS11286-pKPHS2 Δ *oriT*-Vir2	*K. pneumoniae*	32	64	32	32	64	64	1	≤0.5	>128	+	+	$(2.28±1.26)×10^{-5}$
HS11286-pKPHS2Δ*oriT*-V	*K. pneumoniae*	32	64	32	32	64	64	1	≤0.5	>128	+	+	$(7.24±0.82)×10^{-7}$
KP2648H	*K. pneumoniae*	128	>128	32	128	>128	1	0.5	≤0.5	>128	+	+	/
KP2648HΔ*traO*	*K. pneumoniae*	128	>128	32	128	>128	1	0.5	≤0.5	>128	+	+	/
EC600-34	*E. coli*	0.25	0.5	0.25	≤0.125	0.25	1	0.25	≤0.5	≤0.125	-	-	$(7, 54±1.06)×10^{-1}$
J53-34	*E. coli*	0.25	0.5	0.5	0.5	0.5	1	0.25	≤0.5	≤0.125	-	-	$(7, 73±1.91)×10^{-1}$
RJF293-34	*K. pneumoniae*	<0.125	0.5	0.5	0.5	0.5	1	1	≤0.5	≤0.125	+	-	$(5.85±1.35)×10^{-1}$
TC293-34-1	*E. coli*	0.25	0.5	0.5	0.5	0.25	1	0.25	≤0.5	0.5	+	-	
TC293-34-2	*E. coli*	0.5	0.5	0.5	0.5	0.25	1	0.25	≤0.5	0.5	+	-	(1.48±0.73)×10-5
TC293-34-3	*E. coli*	0.5	0.5	0.5	0.25	0.25	1	0.25	≤0.5	0.5	+	-	
HS11286-pKPHS2Δ*oriT*-34	*K. pneumoniae*	16	>32	32	32	64	64	1	≤0.5	>128	-	+	/
TC11286-pKPHS2Δ*oriT*-34	*E. coli*	16	>32	32	32	64	1	0.5	≤0.5	>128	-	+	$(1.49±0.46)×10^{-5}$
KP3038	*K. pneumoniae*	128	>128	128	`128	>128	64	8	≤0.5	>128	-	+	/
KP3038-34	*K. pneumoniae*	64	>128	32	64	>128	64	8	≤0.5	>128	-	+	$(9.77±0.15)×10^{-1}$
TC3038-34	*E. coli*	64	32	128	64	>128	1	0.25	≤0.5	>128	-	+	$(6.02±4.01)×10^{-5}$

Abbreviations: MICs, minimum inhibitory concentration; CAZ, Ceftazidime; ATM, Aztreonam; IPM, Imipenem; MEM, Meropenem; ETP, Ertapenem; AMK, Amikacin; TIG, Tigecycline; CST, Colistin; CTX, Cefotaxime.

Table S2. Strains and plasmids used in this study.

Strains	Species	Feature	Remarks	Reference
KP2648	*K. pneumoniae*	ST11, KL64 serotype, *rmpA*$^+$,*bla* $_{KPC-2}$$^+$	Clinical CRKP strain, harboring three natural plasmids：pKP2648-Vir, pKP2648-KPC and pKP2648-34, Te^R, Mem^R	This study
C600	*E. coli*	T4SS$^-$	Recipient used in the conjugation assay, Rif^R	Appleyard RK. (1)
EC2648-Vir1	*E. coli*	*rmpA*$^+$	Transconjugant derived from C600 and KP2648, harboring pVir-fusion, Rif^R, Te^R	This study
EC2648-Vir2	*E. coli*	*rmpA*$^+$	Transconjugant derived from C600 and KP2648, harboring two plasmids (pKP2648-VirV and pKP2648-34V), Rif^R, Te^R	This study
EC2648-R1	*E. coli*	*bla* $_{KPC-2}$$^+$	Transconjugant derived from C600 and KP2648, harboring pKPC-fusion, Rif^R, Mem^R	This study
EC2648-R1-1	*E. coli*	*bla* $_{KPC-2}$$^+$	Transconjugant derived from C600 and KP2648, harboring pKPC-fusion-1, Rif^R, Mem^R	This study
EC2648-R2	*E. coli*	*bla* $_{KPC-2}$$^+$	Transconjugant derived from C600 and KP2648, harboring two plasmids (pKP2648-KPCR and pKP2648-34R), Rif^R, Mem^R	This study
J53	*E. coli*	T4SS$^-$	Recipient used in the conjugation assay, Az^R	Li, et al. (2)
J-Vir1	*E. coli*	*rmpA*$^+$	Transconjugant derived from J53 and EC2648-Vir1, harboring pVir-fusion, Az^R, Te^R	This study
J-Vir2	*E. coli*	*rmpA*$^+$	Transconjugant derived from J53 and EC2648-Vir2, harboring two plasmids (pKP2648-VirV and pKP2648-34V), Az^R, Te^R	This study
J-R1	*E. coli*	*bla* $_{KPC-2}$$^+$	Transconjugant derived from J53 and EC2648-R1, harboring pKPC-fusion, Az^R, Mem^R	This study
J-R2	*E. coli*	*bla* $_{KPC-2}$$^+$	Transconjugant derived from J53 and EC2648-R2, harboring two plasmids (pKP2648-KPCR and pKP2648-34R), Az^R, Mem^R	This study
RJF293	*K. pneumoniae*	ST374, K2 serotype, *rmpA*$^+$	Clinical hvKP strain harboring a virulence plasmid pRJF293, previously isolated from blood specimen in Ruijin Hospital, Te^R	Wang, et al. (3)
RJF293-R1	*K. pneumoniae*	*rmpA*$^+$,*bla* $_{KPC-2}$$^+$	Transconjugant derived from EC2648-R1 and RJF293 , harboring pRJF293 and pKPC-fusion , Mem^R, Te^R	This study
RJF293-R2	*K. pneumoniae*	*rmpA*$^+$,*bla* $_{KPC-2}$$^+$	Transconjugant derived from EC2648-R2 and RJF293, harboring pRJF293, pKP2648-KPCR and pKP2648-34R, Te^R, Mem^R	This study
RJF999	*K. pneumoniae*	ST23, K1 serotype, *rmpA*$^+$	Clinical hvKP strain harboring a virulence plasmid pRJF999, isolated from blood specimen in Ruijin Hospital, Te^R	Qu, et al. (4)
RJF999-R1	K. pneumoniae	*rmpA*$^+$, *bla* $_{KPC-2}$$^+$	Transconjugant derived from EC2648-R1 and RJF999 , harboring pRJF999 and pKPC-fusion, Mem^R, Te^R	This study
RJF999-R2	*K. pneumoniae*	*rmpA*$^+$, *bla* $_{KPC-2}$$^+$	Transconjugant derived from EC2648-R2 and RJF999, harboring pRJF999,pKP2648-KPCR and pKP2648-34R, Te^R, Mem^R	This study
HS11286	*K. pneumoniae*	ST11, K47 serotype,*bla* $_{KPC-2}$$^+$	Clinical CRKP strain, harboring six plasmids pKHS1-pKHS6, in which the pKPHS2 harboring *bla* $_{KPC-2}$ contains compete T4SS and pKPHS3 contains incompete T4SS, isolated from a sputum specimen at Huashan Hospital, Mem^R	Liu, et al. (5)
HS11286-pKPHS2Δ*oriT*	*K. pneumoniae*	ST11, K47 serotype,*bla* $_{KPC-2}$$^+$	Derivative of HS11286, in which the oriT of pKPHS2 is deficient, Mem^R	Xu, et al. (6)
HS11286-pKPHS2Δ*oriT*-Vir1	*K. pneumoniae*	*bla* $_{KPC-2}$$^+$, *rmpA*$^+$	Transconjugant derived from HS11286-pKPHS2Δ*oriT* and EC2648-Vir1, harboring pKPHS1, pKPHS2Δ*oriT*, pKPHS3-pKPHS6 and pVir-fusion, Mem^R, Te^R	This study
HS11286-pKPHS2Δ*oriT*-Vir2	*K. pneumoniae*	*bla* $_{KPC-2}$$^+$, *rmpA*$^+$	Transconjugant derived from HS11286-pKPHS2Δ*oriT* and EC2648-Vir2, harboring pKPHS1, pKPHS2Δ*oriT*, pKPHS3-pKPHS6, pKP2648-VirV & pKP2648-34V, Mem^R, Te^R	This study
HS11286-pKPHS2Δ*oriT*-V	*K. pneumoniae*	*bla* $_{KPC-2}$$^+$, *rmpA*$^+$	Transconjugant derived from HS11286-pKPHS2Δ*oriT* and KP2648, harboring pKPHS1, pKPHS2Δ*oriT*, pKPHS3-pKPHS6 and pVir-fusion, Mem^R, Te^R	This study
KP2648H	*K. pneumoniae*	*hph*$^+$, *rmpA*$^+$,*bla* $_{KPC-2}$$^+$	Derivative of KP2648, with the insertion of the hygromycin resistant gene hph in pKP2648-34, harboring three plasmids：pKP2648-Vir, pKP2648-KPC and pKP2648-34H, Te^R, Mem^R, Hm^R	This study
KP2648HΔtraO	*K. pneumoniae*	*kana*$^+$, *hph*$^+$, *rmpA*$^+$, *bla* $_{KPC-2}$$^+$	Derivative of KP2648H, with the replacement of traO of pKP2648-34H by kana, harboring three plasmids: pKP2648-34HΔ*traO*, pKP2648-Vir, pKP2648-KPC,Te^R, Mem^R, Hm^R, Kana^R	This study
C600-34	*E. coli*	*hph*$^+$	Transconjugant derived from KP2648Hm and C600, harboring pKP2648-34H, Hm^R, Rif^R	This study
J53-34	*E. coli*	*hph*$^+$	Transconjugant derived from C600-34 and J53, harboring pKP2648-34H, Hm^R, Az^R	This study
RJF293-34	*K. pneumoniae*	*rmpA*$^+$, *hph*$^+$	Transconjugant derived from C600-34 and RJF293, harboring pKP2648-34H and pRJF293 , Te^R, Hm^R	This study
TC293-34-1	*E. coli*	*rmpA*$^+$, *hph*$^+$	Transconjugant derived from RJF293-34 and C600, harboring pRJF293-34fusion, Te^R, Hm^R,	This study
TC293-34-2	*E. coli*	*rmpA*$^+$, *hph*$^+$	Transconjugant derived from RJF293-34 and C600, harboring pRJF293, pKP2648-34H, Te^R, Hm^R, Rif^R	This study
TC293-34-3	*E. coli*	*rmpA*$^+$, *hph*$^+$	Transconjugant derived from RJF293-34 and C600, harboring pRJF293-short, pKP2648-34H, Te^R, Hm^R, Rif^R	This study
HS11286-pKPHS2Δ*oriT*-34	*K. pneumoniae*	*bla* $_{KPC-2}$$^+$, *hph*$^+$	Transconjugant derived from C600-34 and HS11286-pKPHS2ΔoriT, harboring pKP2648-34H, pKPHS1, pKPHS2Δ*oriT*, pKPHS3-pKPHS6 , Mem^R, Hm^R	This study
TC11286-pKPHS2Δ*oriT*-34	*E. coli*	*bla* $_{KPC-2}$$^+$, *hph*$^+$	Transconjugant derived from HS11286-pKPHS2Δ*oriT*-34 and C600, harboring pKPHS2Δ*oriT*-34fusion, Mem^R, Hm^R, Rif^R	This study
KP3038	*K. pneumoniae*	ST11,*bla* $_{KPC-2}$$^+$	Clinical CRKP strain, harboring two plasmids pKP3038-1,pKP3038-2 ,isolated from an anal swab specimen in Ruijin Hospital, Mem^R	This study
KP3038-34	*K. pneumoniae*	*bla* $_{KPC-2}$$^+$, *hph*$^+$	Transconjugant derived from C600-34 and KP3038, harboring pKP2648-34H, pKP3038-1,pKP3038-2, Mem^R, Hm^R	This study
TC3038-34	*E. coli*	*bla* $_{KPC-2}$$^+$, *hph*$^+$	Transconjugant derived from KP3038-34 and C600, harboring pKP3038-1-34fusion， Mem^R, Hm^R, Rif^R	This study

Plasmid	Feature	Remarks	Reference
pKP2648-Vir	IncFIB, IncHI3B, T4SS$^-$, *rmpA*$^+$,Tn*As1*$^-$	Natural virulence plasmid in KP2648,non-conjugative, Te^R	This study
pKP2648-KPC	IncFII(pHN7A8)/IncR, incomplete T4SS, *bla* $_{KPC-}$	Natural resistance plasmid in KP2648,non-conjugative, Mem^R	This study
pKP2648-34	IncN3, T4SS$^+$,Tn*As1*$^-$	Natural plasmid in KP2648,conjugative	This study
pVir-fusion	T4SS$^+$,*rmpA*$^+$	Recombination between pKP2648-Vir and pKP2648-34 in the transconjugant EC2648-Vir1,	This study
pKPC-fusion	T4SS$^+$,*bla* $_{KPC-2}$$^+$	Recombination between pKP2648-KPC and pKP2648-34 in the transconjugant EC2648-R1,	This study
pKPC-fusion-1	incomplete T4SS, *bla* $_{KPC-2}$$^+$	Recombination between pKP2648-KPC and pKP2648-34 in the transconjugant EC2648-R1-1, non-conjugative, Mem^R	This study
pKP2648-VirV	IncFIB, IncHI3B, T4SS$^-$, *rmpA*$^+$,Tn*As1*$^+$	Derivative of pKP2648-Vir in the transconjugant EC2648-Vir2,non-conjugative, Te^R	This study
pKP2648-34V	IncN3, T4SS$^+$,Tn*As1*$^+$	Derivative of pKP2648-34 in the transconjugant EC2648-Vir2,conjugative	This study
pKP2648-KPCR	IncFII(pHN7A8)/IncR, incomplete T4SS, *bla* $_{KPC-}$	Derivative of pKP2648-KPC in the transconjugant EC2648-R2,non-conjugative, Mem^R	This study
pKP2648-34R	IncN3, T4SS$^+$,Tn*As1*$^+$	Derivative of pKP2648-34 in the transconjugant EC2648-R2,conjugative	This study
pRJF293	IncFIB, IncHI3B,T4SS$^-$, *rmpA*$^+$	Natural virulence plasmid in RJF293,non-conjugative, Te^R	Wang, et al. (3)
pRJF999	IncFIB, IncHI3B,T4SS$^-$, *rmpA*$^+$	Natural virulence plasmid in RJF999,non-conjugative, Te^R	Qu, et al. (4)
pKPHS2	IncFII(K)/IncR, T4SS$^+$, *bla* $_{KPC-2}$$^+$	Natural resistance plasmid in HS11286, conjugative, Mem^R	Liu, et al. (5)
pKPHS2Δ*oriT*	oriT$^-$,*bla* $_{KPC-2}$$^+$	Derivative of pKPHS2 derived from CRKP HS11286, *oriT*-deficient,non-conjugative, Mem^R	Xu, et al. (6)
pKP2648-34H	IncN3, T4SS$^+$, *hph*$^+$	Hygromycin resistant derivative of pKP2648-34 derived from KP2648，with the insertion of *hph* in pKP2648-34,conjugative, Hm^R	This study
pKP2648-*34HΔtraO*	IncN3, T4SS$^+$, *hph*$^+$, *traO*$^-$, *kana*$^+$	Derivative of pKP2648-34H, with the replacement of *traO* by kana, non-conjugative, Hm^R,kana^R	This study
pRJF293-34fusion	T4SS$^+$, *rmpA*$^+$, *hph*$^+$	Recombination between pKP2648-34H and pRJF293 in the transconjugant TC293-34-1 ,Te^R,	This study
pRJF293-short	T4SS$^-$, *rmpA*$^+$	Derivative of pRJF293, with a shorten form, Te^R	This study
pKPHS2Δ*oriT*-34fusion	T4SS$^+$, *bla* $_{KPC-2}$$^+$, *hph*$^+$	Recombination between pKP2648-34H andpKPHS2Δ*oriT* in the transconjugant TC293-34-1 ,Te^R, Hm^R	This study
pKP3038-1	T4SS$^-$, *bla* $_{KPC-2}$$^+$	Natural resistance plasmid in KP3038,non-conjugative, Mem^R	This study
pKP3038-1-34fusion	T4SS$^+$,*bla* $_{KPC-2}$$^+$	Recombination between pKP3038-KPC and pKP2648-34 in the transconjugant TC3038-34, Mem^R, Hm^R	This study

Abbreviations: CR-hvKP,Carbapenem-resistant and hypervirulent *Klebsiella pneumoniae* ; hvKP, hypervirulent *Klebsiella pneumoniae* ; Hm^R, hygromycin resistance; Te^R, potassium tellurite resistance; Az^R, sodium azide resistance; Mem^R,meropenem resistance; Rif^R, rifampicin resistance, $Kana^R$, kanamycin resistance.

Table S3. Oligonucleotides used in this study.

Name	Sequence (5'-3')	Description	Source or Reference
**rpoB-F**	GTTTTCCCAGTCACGACGTTGTAGGCGAAATGGCWGAGAACCA	Primers used for identification of MLST	https://bigsdb.pasteur.fr/klebsiella/primers_used.html
**rpoB-R**	TTGTGAGCGGATAACAATTTCGAGTCTTCGAAGTTGTAACC		
**gapA-F**	GTTTTCCCAGTCACGACGTTGTATGAAATATGACTCCACTCACGG		
**gapA-R**	TTGTGAGCGGATAACAATTTCCTTCAGAAGCGGCTTTGATGGCTT		
**mdh-F**	GTTTTCCCAGTCACGACGTTGTA CCCAACTCGCTTCAGGTTCAG		
**mdh-R**	TTGTGAGCGGATAACAATTTCCCGTTTTTCCCCAGCAGCAG		
**pgi-F**	GTTTTCCCAGTCACGACGTTGTAGAGAAAAACCTGCCTGTACTGCTGGC		
**pgi-R**	TTGTGAGCGGATAACAATTTCCGCGCCACGCTTTATAGCGGTTAAT		
**phoE-F**	GTTTTCCCAGTCACGACGTTGTAACCTACCGCAACACCGACTTCTTCGG		
**phoE-R**	TTGTGAGCGGATAACAATTTCTGATCAGAACTGGTAGGTGAT		
**infB-F**	GTTTTCCCAGTCACGACGTTGTACTCGCTGCTGGACTATATTCG		
**infB-R**	TTGTGAGCGGATAACAATTTC CGCTTTCAGCTCAAGAACTTC		
**tonB-F**	GTTTTCCCAGTCACGACGTTGTACTTTATACCTCGGTACATCAGGTT		
**tonB-R**	TTGTGAGCGGATAACAATTTCATTCGCCGGCTGRGCRGAGAG		
**KPC-F**	CGTCTAGTTCTGCTGTCTTG	Detection of carbapenemase resistance genes	（7）
**KPC-R**	CTTGTCATCCTTGTTAGGCG		
**NDM-F**	GGTTTGGCGATCTGGTTTTC		
**NDM-R**	CGGAATGGCTCATCACGATC		
**OXA48-F**	GCGTGGTTAAGGATGAACAC		
**OXA48-R**	CATCAAGTTCAACCCAACCG		
**IMP-F**	GGAATAGAGTGGCTTAAYTCTC		（8）
**IMP-R**	GGTTTAAYAAAACAACCACC		
**VIM-F**	GATGGTGTTTGGTCGCATA		
**VIM-R**	CGAATGCGCAGCACCAG		
**iucA-F**	AATCAATGGCTATTCCCGCTG	Detection of virulence genes	（9）
**iucA-R**	CGCTTCACTTCTTTCACTGACAGG		
**rmpA-F**	ACTGGGCTACCTCTGCTTCA		
**rmpA-R**	CTTGCATGAGCCATCTTTCA		
**peg344-F**	CTTGAAACTATCCCTCCAGTC		
**peg344-F**	CCAGCGAAAGAATAACCCC		
**OriT2_286-01**	CCCaAGCTTATGCCGCGTCGCTTGATCCTC	Amplifying homologous arms of *oriT* region for replacement of *oriT* on pKPHS2 with hygromycin resistance cassette containing FRT sites	（6）
**OriT2_286-02**	TAGTACCATACAATGCTGCACAGT		
**OriT2_286-03**	AAACACTGTGCAGCATTGTATGGT		
**OriT2_286-04**	TAGTAAGGCTGCAAGTCTTGAAAA		
**OriT2_286-DF**	AAACTTTTCAAGACTTGCAGCCTT	Detecting the *oriT* region on pKPHS2 or its derivatives	
**OriT2_286-DR**	GTGATGCCGATTATTATCCAGG		
**Hm-F**	TATAACTCCTGGCCGATCGAATTAGCTTCA	Amplifying hygromycin resistance cassette	This study
**Hm-R**	ATATCAGAATAAAAATTAAACGTCCTTAAT		This study
**T34-01**	TTCCTTCGCAGTTTGCCCGG	Adding a hygromycin resistance gene on the pKP2648-34	This study
**T34-02/HmF**	TCGATCGGCCAGGAGTTATATAAATGGCAA		This study
**T34-03/HmR**	TCCCCAATTAATTAAGGACGTTTAATTTTT		This study
**T34-04**	GCAGACTGAATGGCATCGCG		This study
**hph-DF**	TATTCAAAAGCGTGCGCCGG	Detecting hygromycin resistance gene added on pKP2648-34 correctly	This study
**hph-DR**	CTTGACCTGCGGGGCTTTTA		This study
**kana-F**	GAGGCTGATCTAGGCTGGAGCTGCTTCGAA	Amplifying kanamycin resistance cassette	This study
**kana-R**	CTGTCTTTTCTGGTTGGTCCATATGAATAT		This study
**TraO_34-01**	CGCGGGAAGATCTGAATAAG	Amplifying homologous arms for replacement on *traO* gene on pKP2648-34 with kanamycin resistance cassette	This study
**TraO_34-02/kana**	CTCCAGCCTAGATCAGCCTCCCACTGCGCC		This study
**TraO_34-03/kana**	GGACCAACCAGAAAAGACAGAGAGGAAAAC		This study
**TraO_34-04**	TCAGCAGAGTACACATCACG		This study
**TraO-DF**	TGTATCGTGCAGATGCCGCT	Detecting *traO* gene on pKP2648-34 or its derivatives	This study
**TraO-DR**	TCAGCAGAGTACACATCACG		This study
**iucD-F**	AGGCGTGAAGTATTCGTTGG	Detecting specific backbone genes on pKP2648-Vir	This study
**iucD-R**	ACCGAACTGCGTACCGTATC		This study
**iucC-F**	AAGGTAATTCGGCAACATGC		This study
**iucC-R**	GTGGTTCCGCTGTATCACCT		This study
**iucB-F**	CGCTTTGCTCCCAGAAATAC		This study
**iucB-R**	CGCTGTGAAAAACTCGACAA		This study
**iucA-F**	ATAAGGGAAGTAGCGCAGCA		This study
**iucA-R**	GTGAAAACCTGCTGGTGGAT		This study
**HNS-F**	GTACCGGGAAATGATGATCG		This study
**HNS-R**	TTGCTCACCAGCTTCGTCTA		This study
**iutA-F**	GACGAAGAGAAAGCCGTCAC		This study
**iutA-R**	ACGTCTTTCCGTGGCATATC		This study
**rmpA-F**	GGAAATGGGGAGGGTACAAA		This study
**rmpA-R**	GCATGAGCCATCTTTCATCA		This study
**TrbB**	CATAGAGCGCAACGAAAACA		This study
**TrbB**	GGCCTTTCGTTTCTGTTTCA		This study
**IncFIB-F**	GACACCCTGGAACTGTCGTT		This study
**IncFIB-F**	TAGGCTGACTGCACCAGATG		This study
**IncHI3B-F**	AGCACACCCATTTCCAGTTC		This study
**IncHI3B-R**	CTCATCGGTTCCCTCATCAT		This study
**SHV-F**	AGCCGCTTGAGCAAATTAAA		This study
**SHV-R**	GCCTCATTCAGTTCCGTTTC		This study
**CTX-M-F**	CGGCTGGGTAAAATAGGTCA		This study
**CTX-M-R**	CGTTGCAGTACAGCGACAAT		This study
**IncR-F**	TTTACCCCAGCCTTCACATC		This study
**IncR-R**	ACGACAGCCAGGAAGATGAC		This study

**IncFⅡ-F**	CTTGAACTTTTCCGGGCATA		This study
**IncFⅡ-R**	AGCGAAAACCCCGATAATCT		This study
**traA-F**	AGAACCACCCATTTGACGAC	Detecting specific backborn genes on pKP2648-KPC	This study
**traA-R**	AGTGTTCAGGGCTCTGCTGT		This study
**traN-F**	CTGCAGCTCATCAACCGTAA		This study
**traN-R**	TGATGTCTTCTGCCTTGACG		This study
**traG-F**	AGTACGGGATGCCATTTCAG		This study
**traG-R**	CAGTAATGAGCAGGCGACAA		This study
**traM-F**	CTCGGGGAAGAAGTGAGAAA		This study
**traM-R**	GGCAAAGGAAAAGGATGTCA		This study
**traX-F**	GTTAAGCGCCGGAATAACAG		This study
**traX-R**	AGCGGGATATCATCAAAACG		This study
**N3-F**	TTTTCTACCTTCGCCTGTGG		This study
**N3-R**	GTGCAAACGAGTTTCTGCAA		This study
**34-F**	GATACCCTGGCCTTTTAGCC		This study
**34-R**	TTGACGAAGCAGGGGTAATC		This study
**trwN-F**	CAAAATGGCTAACGGCTCAT		This study
**trwN-R**	GTCTTTATCCGAGCCACCAA		This study
**trwM-F**	GGTGTTCCGCGAACAGTAAT		This study
**trwM-R**	AACCACAGCAAGCCAAAAAG	Detecting specific backborn genes on pKP2648-34	This study
**TrwJ-F**	CGCTTGAGCAGGTCACATTA		This study
**TrwJ-R**	CCTGAAGGTCAGCGATTTGT		This study
**TewH-F**	AATACACGGCAATGGCACTT		This study
**TewH-R**	CGGCGGGATACGTCTTATTA		This study
**TrwF-F**	CGCCGTGGAGTATGAAAAAT		This study
**TrwF-R**	TTTCACGCGCTTAACATCTG		This study
**TrwD-F**	TTCAGTGCTTCACGCATTTC		This study
**TrwD-R**	CCAGAACAGAAGGGTGGGTA		This study

Abbreviations: MLST, Multilocus Sequence Typing

Table S4. Antibiotics and the corresponding concentrations used for each conjugation pair.

Plasmid	Donor	Recipient	Antibiotic and antibiotic concentration
Transferability of virulence plasmid pKP2648-Vir	KP2648	C600	2 μg/ml K_2TeO_3 + 100 μg/ml rifampicin
	EC2648-Vir1	J53	2 μg/ml K2TeO3 + 100 μg/ml sodium azide
	EC2648-Vir2	J53	2 μg/ml K2TeO3 + 100 μg/ml sodium azide
	EC2648-Vir1	HS11286-pKPHS2Δ*oriT*	2 μg/ml K_2TeO_3 + 2 μg/ml meropenem
	EC2648-Vir2	HS11286-pKPHS2Δ*oriT*	2 μg/ml K_2TeO_3 + 2 μg/ml meropenem
	KP2648H	C600	2 μg/ml K_2TeO_3 + 100 μg/ml rifampicin
	KP2648	HS11286-pKPHS2Δ*oriT*	2 μg/ml K_2TeO_3 + 200 μg/ml hygromycin B
	KP2648HΔ*traO* (failure)	C600	2 μg/ml K_2TeO_3 + 100 μg/ml rifampicin
Transferability of resistance plasmid pKP2648-KPC	KP2648	C600	2 μg/ml meropenem + 100 μg/ml rifampicin
	EC2648-R1	J53	2 μg/ml meropenem + 100 μg/ml sodium azide
	EC2648-R1-1(failure)	J53	2 μg/ml meropenem + 100 μg/ml sodium azide
	EC2648-R2	J53	2 μg/ml meropenem + 100 μg/ml sodium azide
	EC2648-R1	RJF293	2 μg/ml meropenem + 2 μg/ml K2TeO3
	EC2648-R1-1(failure)	RJF293	2 μg/ml meropenem + 2 μg/ml K2TeO3
	EC2648-R2	RJF293	2 μg/ml meropenem + 2 μg/ml K2TeO3
	EC2648-R1	RJF999	2 μg/ml meropenem + 2 μg/ml K2TeO3
	EC2648-R1-1(failure)	RJF999	2 μg/ml meropenem + 2 μg/ml K2TeO3
	EC2648-R2	RJF999	2 μg/ml meropenem + 2 μg/ml K2TeO3
	KP2648H	C600	2 μg/ml meropenem + 100 μg/ml rifampicin
	KP2648(failure)	RJF293H*	2 μg/ml meropenem + 200 μg/ml hygromycin B
	KP2648(failure)	RJF293ZH*	2 μg/ml meropenem + 200 μg/ml hygromycin B
	KP2648HΔ*traO* (failure)	C600	2 μg/ml meropenem + 100 μg/ml rifampicin
Co-transfer of virulence plasmid pKP2648-Vir and resistance plasmid pKP2648-KPC	KP2648(failure)	C600	2 μg/ml K_2TeO_3 +2 μg/ml meropenem + 100 μg/ml rifampicin
Transferability of conjugative helper plasmid pKP2648-34H	KP2648H	C600	200 μg/ml hygromycin B + 100 μg/ml rifampicin
	C600-34	J53	200 μg/ml hygromycin B + 100 μg/ml sodium azide
	C600-34	RJF293	200 μg/ml hygromycin B + 2 μg/ml K2TeO3
	C600-34	KP3038	200 μg/ml hygromycin B + 2 μg/ml meropenem
	C600-34	HS11286-pKPHS2Δ*oriT*	200 μg/ml hygromycin B + 2 μg/ml meropenem
Transferability of pKP2648-34HΔ	KP2648HΔ*traO* (failure)	C600	200 μg/ml hygromycin B + 100 μg/ml rifampicin
Transferability of virulence plasmid pRJF293	RJF293(failure)	C600	2 μg/ml K_2TeO_3 + 100 μg/ml rifampicin
	RJF293-34	C600	2 μg/ml K_2TeO_3 + 100 μg/ml rifampicin
Transferability of resistance plasmid pKP3038-KPC	KP3038(failure)	C600	2 μg/ml meropenem + 100 μg/ml rifampicin
	KP3038-34	C600	2 μg/ml meropenem + 100 μg/ml rifampicin
Transferability of resistance plasmid pKPHS2Δ*oriT*	HS11286-pKPHS2Δ *oriT* (failure)	C600	2 μg/ml meropenem + 100 μg/ml rifampicin
	HS11286-pKPHS2Δ*oriT*-34	C600	2 μg/ml meropenem + 100 μg/ml rifampicin

* RJF293H is the derivative of RJF293, with the insertion of the hygromycin resistant gene *hph* in the chromosome. *rmpA* $^+$, *hph* $^+$,hypermucoviscous

* RJF293ZH is the derivative of RJF293H, with the knockout of the rmpA gene in the virulence plasmid. *rmpA* $^-$, *hph* $^+$, hypomucoviscous

Reference

1.Appleyard RK. 1954. Segregation of New Lysogenic Types during Growth of a Doubly Lysogenic Strain Derived from Escherichia Coli K12. Genetics 39:440-52.

2. Li G, Wei Q, Wang Y, Du X, Zhao Y, Jiang X. 2011. Novel genetic environment of the plasmid-mediated KPC-3 gene detected in Escherichia coli and Citrobacter freundii isolates from China. Eur J Clin Microbiol Infect Dis 30:575-80.

3. Wang XL, Xie YZ, Li G, Liu JL, Li XB, Tian LJ, Sun J, Ou HY, Qu HP. 2018. Whole-Genome-Sequencing characterization of bloodstream infection-causing hypervirulent Klebsiella pneumoniae of capsular serotype K2 and ST374. Virulence 9:510-521.

4. Qu,H. and Ou,H.-Y. Accession: PRJNA307276.

5.Liu P, Li P, Jiang X, Bi D, Xie Y, Tai C, Deng Z, Rajakumar K, Ou HY. 2012. Complete genome sequence of Klebsiella pneumoniae subsp. pneumoniae HS11286, a multidrug-resistant strain isolated from human sputum. J Bacteriol 194:1841-2.

6. Xu YP, Zhang JF, Wang M, Liu M, Liu GT, Qu HP, Liu JL, Deng ZX, Sun JY, Ou HY, Qu JM. 2021. Mobilization of the nonconjugative virulence plasmid from hypervirulent Klebsiella pneumoniae. Genome Medicine 13.

7. Poirel L, Walsh TR, Cuvillier V, Nordmann P. 2011. Multiplex PCR for detection of acquired carbapenemase genes. Diagn Microbiol Infect Dis 70:119-23.

8. Ellington MJ, Kistler J, Livermore DM, Woodford N. 2007. Multiplex PCR for rapid detection of genes encoding acquired metallo-beta-lactamases. J Antimicrob Chemother 59:321-2.

9. Liu C, Du P, Xiao N, Ji F, Russo TA, Guo J. 2020. Hypervirulent Klebsiella

pneumoniae is emerging as an increasingly prevalent K. pneumoniae pathotype responsible for nosocomial and healthcare-associated infections in Beijing, China. Virulence 11:1215-1224.
